# Revealing the global emission gaps for fully fluorinated greenhouse gases

**DOI:** 10.1038/s41598-024-58504-x

**Published:** 2024-04-16

**Authors:** Liya Guo, Xuekun Fang

**Affiliations:** 1 College of Environmental and Resource Sciences, Zhejiang University, Hangzhou, 310058 Zhejiang China; 2 Center for Global Change Science, Massachusetts Institute of Technology, Cambridge, MA 02139 USA

**Keywords:** Climate sciences, Environmental sciences

## Abstract

In response to the global trend of climate change, it is important to accurately quantify emissions of fully fluorinated greenhouse gases (FFGHGs, referring to SF_6_/NF_3_/CF_4_/C_2_F_6_/C_3_F_8_/*c*-C_4_F_8_ here). Atmospheric observation-based top-down methods and activity-based bottom-up methods are usually used together to estimate FFGHG emissions at the global and regional levels. In this work, emission gaps at global and regional levels are discussed among top-down studies, between the top-down and bottom-up FFGHG emissions, and among bottom-up emissions. Generally, trends and magnitudes of individual FFGHG emissions among top-down estimates are close to each other within the uncertainties. However, global bottom-up inventories show discrepancies in FFGHG emissions among each other in trends and magnitudes. The differences in emission magnitudes are up to 93%, 90%, 88%, 83%, 87%, and 85% for SF_6_, NF_3_, CF_4_, C_2_F_6_, C_3_F_8_, and *c*-C_4_F_8_, respectively. Besides, we reveal the insufficient regional TD studies and the lack of atmospheric observation data/stations especially in areas with potential FFGHG emissions. We make recommendations regarding the best practices for improving our understanding of these emissions, including both top-down and bottom-up methods.

## Introduction

Human-made fully fluorinated greenhouse gases (FFGHGs), covering Sulfur Hexafluoride (SF_6_), Nitrogen Trifluoride (NF_3_), and Perfluorocarbons (PFCs, including CF_4_, C_2_F_6_, C_3_F_8_, and *c*-C_4_F_8_ here) are almost emitted from various industrial processes and product use, such as electrical equipment^1–3^, primary aluminum production^4–6^, and semiconductor manufacturing^4,6,7^. They are significant because of their lifetime of hundreds to thousands of years and high global warming potentials over a 100-years horizon (GWP_100_) (Supplementary Table 1). They have been regulated under the Kyoto Protocol (KP)^8^ and the subsequent Doha amendment^9^ (adding NF_3_ in this amendment) as well as the Paris Agreement (PA)^10^ under the United Nations Framework Convention on Climate Change (UNFCCC). 

FFGHG emissions are estimated by two methods: bottom-up (BU) and top-down (TD) methods. The BU method calculates the annual sectoral FFGHG emissions by activity data and emission factors. The TD method uses the observed atmospheric concentrations of FFGHGs and an atmospheric model to evaluate the FFGHG emissions. By contrast, using the BU method, several inventory sources including the Emissions Database for Global Atmospheric Research (EDGAR), emissions submitted to the UNFCCC (abbreviated as “UNFCCC” afterward), and the Environmental Protection Agency (EPA) in the United States (US) have reported the FFGHG emissions from individual countries including the Annex I countries (mainly developed countries) and non-Annex I countries (developing countries). Thus, in this work, the term “inventory” will be used to refer to BU estimates only. The atmospheric measurements are usually conducted by networks like the US National Oceanographic and Atmospheric Administration (NOAA), the Advanced Global Atmospheric Gases Experiment (AGAGE) international consortium^11^, and the National Institute for Environmental Studies (NIES)^12^. The quality assurance guidance laid out in the 2019 Refinement to the 2006 IPCC Guidelines for National Greenhouse Gas Inventories states that “Atmospheric measurements are being used to provide useful quality assurance of the national greenhouse gas emission estimates. Under the right measurement and modeling conditions, they can provide a perspective on the trends and magnitude of greenhouse gas (GHG) emission estimates that is largely independent of inventories”^13^. Complementing each other, TD and BU results could provide a better understanding of global and regional FFGHG emissions, and thus contribute to the FFGHG mitigation globally and regionally.

Supplementary Table 2 and Supplementary Table 3 have summarized the previous individual global and regional TD studies on FFGHG emission estimates as much as possible. Available BU estimates from the EDGAR, UNFCCC, and EPA are also used here to understand FFGHG emissions. Taken together, previous TD and BU studies have reported FFGHG emissions ranging from 1900 to 2021. What we want to understand is, (1) whether TD and BU estimates are both available at the global and regional levels or not; (2) whether there is an agreement on the magnitude and trend of FFGHG emissions among different studies at the global and regional levels or not. If not, we want to know more about what the potential deficiencies are and what could be done to improve the accuracy of FFGHG emissions in the future. However, no comprehensive study of the SF_6_/NF_3_/CF_4_/C_2_F_6_/C_3_F_8_/*c*-C_4_F_8_ emission analysis has yet been made to reveal its potential emission gap and pinpoint the common problems of emission quantification. Therefore, we comprehensively collect the global and regional SF_6_/NF_3_/CF_4_/C_2_F_6_/C_3_F_8_/*c*-C_4_F_8_ emissions during 1900–2021 from previous TD and BU studies. Then we systematically compare both the global and regional SF_6_/NF_3_/CF_4_/C_2_F_6_/C_3_F_8_/*c*-C_4_F_8_ emissions across three aspects (TD vs TD, BU vs BU, and TD vs BU) to try to respond to our concerns in this study. Our work would bring a comprehensive perspective on the current state and future direction of FFGHG emission quantification, which is conducive to further promoting the accurate quantification of FFGHG emissions, ultimately serving the FFGHGs mitigation and climate change response.

## Results and discussion

In this work, we compared the FFGHG emissions from TD and BU results (UNFCCC, EDGAR, and EPA) at the global and regional levels. The analysis of FFGHG emission gaps is developed from the following aspects: (1) emission gap among TD at the global level; (2) emission gap among inventories at the global level; (3) emission gap between TD and BU at the global level; (4) emission gap among TD at the regional level; (5) emission gap among inventories at the regional level; (6) emission gap between TD and BU at the regional level. The gaps are reflected in the following two aspects: (a) differences in emission trend; (b) differences in emission magnitude. The detailed information and discussion are shown in the following from global and regional perspectives.

### Emission gap among TD from a global perspective

**Figure 1:**
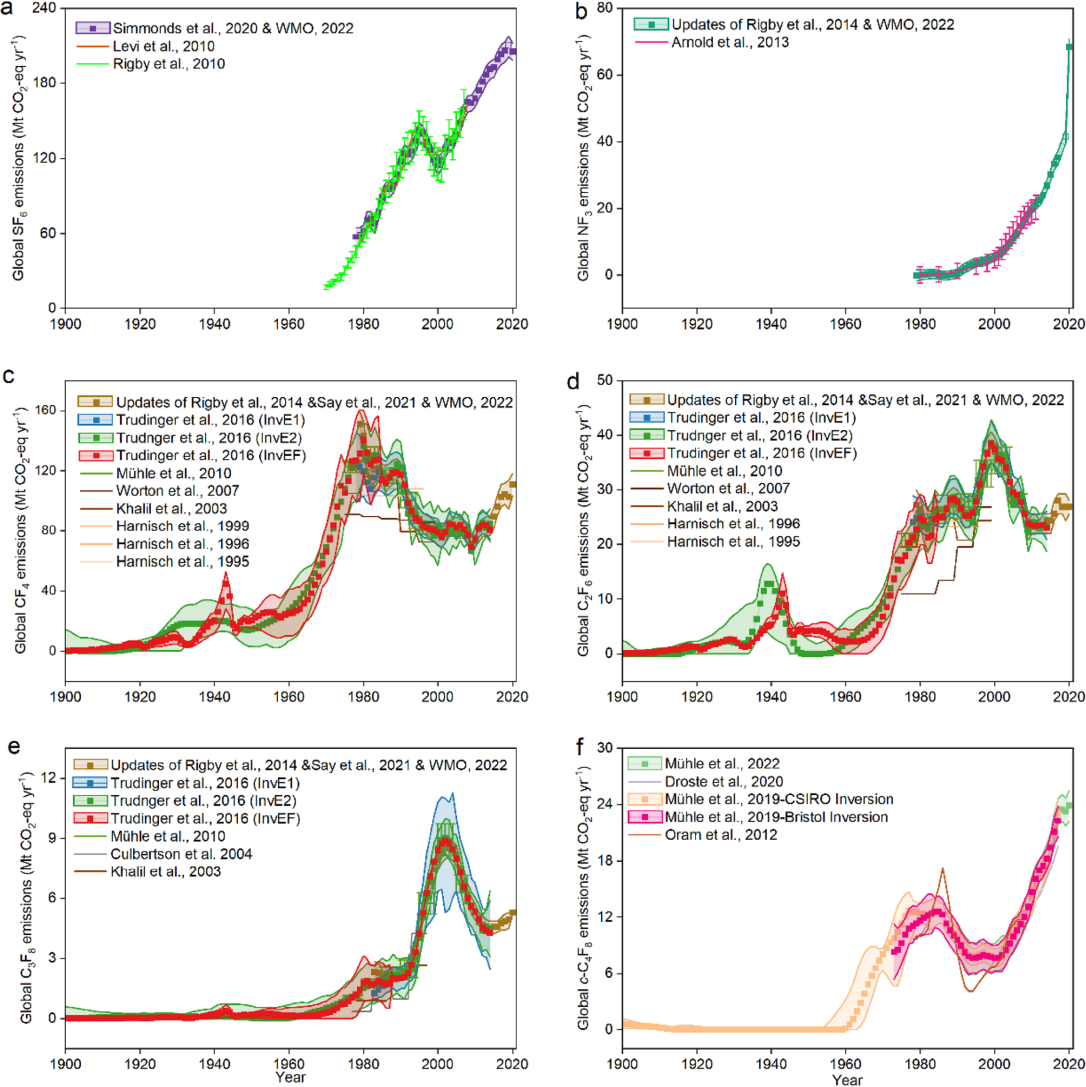
TD global FFGHG CO_2_-equivalent emissions for the individual FFGHG from previous studies for (**a**) SF_6_, (**b**) NF_3_, (**c**) CF_4_, (**d**) C_2_F_6_, (**e**) C_3_F_8_, and (**f**) *c*-C_4_F_8_. Unit: million tons CO_2_-equivalent per year (Mt CO_2_-eq yr^−1^). The detailed sources are listed in Supplementary Table 2. For SF_6_, Simmonds et al., 2020^1^ and WMO, 2022^18^ provided emissions from 1978 to 2018 and 2020. The hollow square means that the SF_6_ emission in 2019 was extrapolated from the recent 5 years’ emissions in Simmonds et al., 2020^1^. For NF_3_, updates of Rigby et al., 2014^16^ and WMO, 2022^18^ provided emissions from 1979 to 2017 and 2020, respectively. The hollow squares mean that the NF_3_ emissions in 2018 and 2019 were extrapolated from the recent 5 years’ emissions in updates of Rigby et al., 2014^16^. For CF_4_, C_2_F_6_, and C_3_F_8_, updates pf Rigby et al., 2014^16^, Say et al., 2021^19^, and WMO, 2022^18^ provided emissions before 2004, from 2005 to 2019, and from 2020, respectively.

Only the global TD emissions of the individual FFGHG from previous works have been summarized in Fig. 1 and Supplementary Table 2. Figure 1a shows that the mean global SF_6_ emissions have increased from 57 Mt CO_2_-eq yr^−1^ in 1978, reaching a peak (143 Mt CO_2_-eq yr^−1^) in about 1995, then decreased to approximately 118 Mt CO_2_-eq yr^−1^ in 2000 or so, again consistently rising to 211 Mt CO_2_-eq yr^−1^ in 2019, followed by a slight decline to 205 Mt CO_2_-eq yr^−1^ in 2020. During 1978–2008, global SF_6_ emissions from three TD studies^1,14,15^ showed similar trends as described above and magnitudes with a mean emission of 112 Mt CO_2_-eq yr^−1^ over this period. Figure 1b shows the significant rise in NF_3_ emissions ranging from 0 in 1979 to 68 Mt CO_2_-eq yr^−1^ in 2020. Throughout 2000–2011, there are consistently NF_3_ increasing trends (increasing rate of 1.4 Mt CO_2_-eq yr^−2^) and magnitudes (average emission of about 12.8 Mt CO_2_-eq yr^−1^) among previous global TD results^16–18^.

Figure 1c illustrates that global CF_4_ emissions^16,18,19^ with fluctuation have grown from 0 in 1900 to 111 Mt CO_2_-eq yr^−1^ in 2020. Note that the global CF_4_ emissions among different studies were relatively close within the uncertainties of TD results. For example, the average global CF_4_ emissions throughout 1900–1978 from Trudinger et al., 2016^20^ using InvE2 and InvEF inversions are both 25 Mt CO_2_-eq yr^−1^. Besides, the average global CF_4_ emissions during 1979–2014 from Trudinger et al., 2016^20^ using InvE1, InvE2, and InvEF inversions are in the range of 94–97 Mt CO_2_-eq yr^−1^, close to the values of other works^16,19^ (97 Mt CO_2_-eq yr^−1^) over the same period. However, the average global CF_4_ emissions (89 Mt CO_2_-eq yr^−1^) over 1975–1989 from Worton et al., 2007^21^ were significantly lower than those from all other global TD studies (103–140 Mt CO_2_-eq yr^−1^) over the same period. Figure 1d illustrates that the global C_2_F_6_ emissions^16,18,19^ vary ranging from 0.037 Mt CO_2_-eq yr^−1^ in 1900 to 27 Mt CO_2_-eq yr^−1^ in 2020. Similarly, the global C_2_F_6_ emissions among different studies during 1900–1978 and 1979–2014 were both relatively close. The average global C_2_F_6_ emissions throughout 1900–1978 from Trudinger et al., 2016^20^ using InvE2 and InvEF inversions are both 4 Mt CO_2_-eq yr^−1^. During 1979–2014, the average global C_2_F_6_ emissions from Trudinger et al., 2016^20^ using InvE1, InvE2, and InvEF inversions are close to 28 Mt CO_2_-eq yr^−1^, consistent with values of other works^16,19^ (28 Mt CO_2_-eq yr^−1^) over this period. However, the average global C_2_F_6_ emissions over 1975–1994 from Worton et al., 2007^21^ were only 14 Mt CO_2_-eq yr^−1^, about half the values (21–26 Mt CO_2_-eq yr^−1^) from all other studies. The choice of an inversion model may cause the CF_4_&C_2_F_6_ emission difference between Worton et al., 2007^21^ and the other TD studies. In Worton’s work, collecting the firn air samples, they used an iterative approach with a firn physical transport model to obtain emissions. While AGEGE 12-box atmospheric transport model^19,22^ or the combination of AGEGE 12-box atmospheric transport model with the iterative approach^20^ were used in other TD studies.

Figure 1e indicates that the global C_3_F_8_ emissions^16,18,19^ ranged from 0.0012 Mt CO_2_-eq yr^−1^ in 1990 to 5.3 Mt CO_2_-eq yr^−1^ in 2020 with a peak in 2003 or so. There is an agreement on the global C_3_F_8_ emission from different TD studies during 1900–1982 (average value of 0.24 Mt CO_2_-eq yr^−1^)^20^ and during 1983–2014 (average value of 5.0 Mt CO_2_-eq yr^−1^)^16,19,20,22^. In Fig. 1f., *c*-C_4_F_8_ emissions^23,24^ rose from 0.61 Mt CO_2_-eq yr^−1^ in 1990 to 23.9 Mt CO_2_-eq yr^−1^ in 2020 with fluctuation over this period. Before 2000, the global *c*-C_4_F_8_ emissions among previous TD studies were close to each other with a similar average emission of 8–10 Mt CO_2_-eq yr^−1^ although the global *c*-C_4_F_8_ emissions from Oram et al., 2012^25^ showed larger variability. After 2000, the global *c*-C_4_F_8_ emissions among previous TD studies were close to each other with a similar average emission of 12–14 Mt CO_2_-eq yr^−1^ and an increasing rate of 0.72–0.96 Mt CO_2_-eq yr^−2^. However, some slight emission gaps for global *c*-C_4_F_8_ results were found before 2000. The average *c*-C_4_F_8_ emissions from Droste et al., 2020^26^ (15 Mt CO_2_-eq yr^−1^ over 1985–1988) and Oram et al., 2012^25^ (15 Mt CO_2_-eq yr^−1^ over 1985–1988) were slightly higher than those from Mühle et al., 2019^24^ [12 Mt CO_2_-eq yr^−1^ over 1985–1988 in CSIRO Inversion; 12 Mt CO_2_-eq yr^−1^ over 1985–1988 in Bristol Inversion], while *c*-C_4_F_8_ emissions (1991–1998) from Droste et al., 2020^26^ (5 Mt CO_2_-eq yr^−1^) and Oram et al., 2012^25^ (5 Mt CO_2_-eq yr^−1^) were slightly lower than results of around 8 Mt CO_2_-eq yr^−1^ from both Mühle et al., 2019^24^ and Mühle et al., 2022^23^. The *c*-C_4_F_8_ datasets from more than one station (including Zeppelin, Mace Head, Jungfraujoch, Monte Cimone, Trinidad Head, Shangdianzi, Gosan, La Jolla, Ragged Point and so on) were employed in other studies^23,24^ to derive *c*-C_4_F_8_ emissions, while Oram et al., 2012^25^ only used the *c*-C_4_F_8_ dataset from Cape Grim to obtain the global TD *c*-C_4_F_8_ emissions, which may explain the larger variability of its results.

### Emission gap among BU from a global perspective

**Figure 2:**
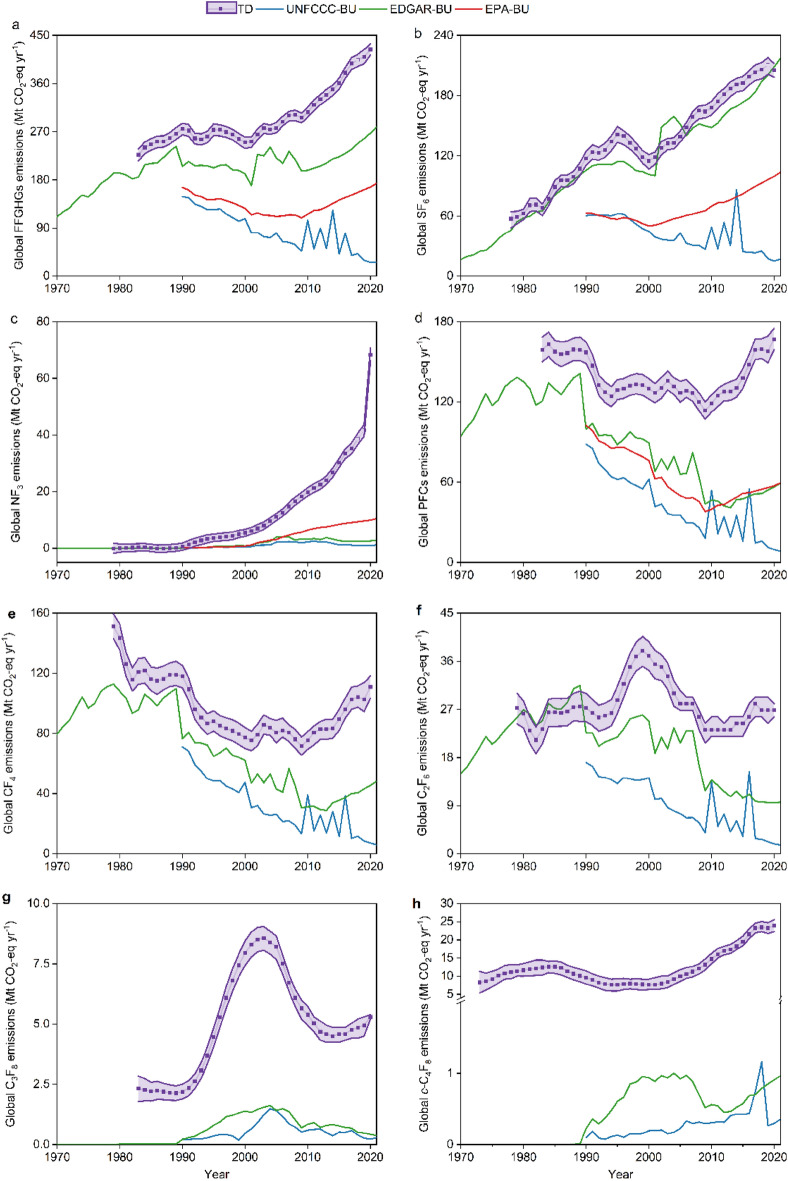
Comparison of FFGHG CO_2_-equivalent emissions from TD and BU on the global scale from 1970 to 2021 for (**a**) total FFGHGs, (**b**) SF_6_, (**c**) NF_3_, (**d**) total PFCs, (**e**) CF_4_, (**f**) C_2_F_6_, (**g**) C_3_F_8_, and (**h**) *c*-C_4_F_8_. Unit: million tons CO_2_-equivalent per year (Mt CO_2_-eq yr^−1^).The global TD emissions of individual FFGHG were from Simmonds et al., 2020^1^ (SF_6_ emissions from 1978 to 2018), Say et al., 2021^19^(CF_4_/C_2_F_6_/C_3_F_8_ emissions from 2005 to 2019), Mühle et al., 2019&2022^23,24^(*c*-C_4_F_8_ emissions from 1973 to 1989^15^ and from 1990 to 2020^27^), updates of Rigby et al., 2014^16^ (NF_3_ emissions from 1979 to 2017 and CF_4_/C_2_F_6_/C_3_F_8_ emissions before 2005), and WMO, 2022^18^ (SF_6_/NF_3_/CF_4_/C_2_F_6_/C_3_F_8_ emissions in 2020). The annual global TD total FFGHG emissions were the sum of six FFGHG global TD emissions. The purple shading area represents the 16th–84th percentile range from the AGAGE 12-box model. The hollow squares mean that these values were extrapolated from the recent 5 years’ emissions. FFGHG emissions in the United Nations Framework Convention on Climate Change (UNFCCC) are obtained from the following website: https://di.unfccc.int/flex_annex1 and https://di.unfccc.int/flex_non_annex1. FFGHG emissions in the Emissions Database for Global Atmospheric Research (EDGAR) are from EDGAR v4.2^37^ (1970–1989) and EDGAR v7.0^38^ (1990–2021). FFGHG CO_2_-equivalent emissions in US Environmental Protection Agency (EPA) were from Global Non-CO_2_ Greenhouse Gas Emission Projections & Marginal Abatement Cost Analysis: Methodology Documentation^39^. Note that EPA only provided total PFC emissions instead of individual PFC emissions. All TD and BU data is accessed before 2023-11-10.

Note that there is a notable difference among these inventory results (Fig. 2). Figure 2a shows that among three inventories, the EDGAR inventory reported the highest global total FFGHG emissions rising from 185 Mt CO_2_-eq yr^−1^ in 1983 to 279 Mt CO_2_-eq yr^−1^ in 2021 (green solid line). However, global total FFGHG emissions reported by EPA kept relatively stable but with a lower magnitude (166 Mt CO_2_-eq yr^−1^ in 1990 to 174 Mt CO_2_-eq yr^−1^ in 2021) (red solid line). The global total FFGHG emissions from UNFCCC dropped from 149 Mt CO_2_-eq yr^−1^ (1990) to 26 Mt CO_2_-eq yr^−1^ (2021) (blue solid line).

Figure 2b–h illustrates the discrepancies among inventories for individual FFGHG. The global SF_6_ emissions reported by the EDGAR rose from 17 Mt CO_2_-eq yr^−1^ to 217 Mt CO_2_-eq yr^−1^ from 1970 to 2021 (Fig. 2b). Despite the same increasing trend with the EDGAR, EPA reported the global SF_6_ emissions with a lower magnitude (63 Mt CO_2_-eq yr^−1^–104 Mt CO_2_-eq yr^−1^ from 1990 to 2021) (Fig. 2b). However, the global SF_6_ emissions submitted to the UNFCCC declined from 60 Mt CO_2_-eq yr^−1^ in 1990 to 17 Mt CO_2_-eq yr^−1^ in 2021 (Fig. 2b). In Fig. 2c, EPA has the highest global NF_3_ emissions with an average of 6 Mt CO_2_-eq yr^−1^ (1.5 Mt CO_2_-eq yr^−1^ for UNFCCC and 2.7 Mt CO_2_-eq yr^−1^ for EDGAR) and shows the highest increase with the rate of 0.46 Mt CO_2_-eq yr^−2^ (0.026 Mt CO_2_-eq yr^−2^ for UNFCCC and 0.093 Mt CO_2_-eq yr^−2^ for EDGAR) over 2000–2021 among the three inventories. In addition, global NF_3_ emissions reported by the EDGAR (0 in 1970 to 2.8 Mt CO_2_-eq yr^−1^ in 2021) display a similar trend to the UNFCCC results (0.10 Mt CO_2_-eq yr^−1^ in 1990 to 1.1 Mt CO_2_-eq yr^−1^ in 2021) but with a slightly higher emission magnitude. For PFCs **(**Fig. 2d**)**, EDGAR and EPA show relatively similar emission trends (− 1.3 Mt CO_2_-eq yr^−2^ for both EDGAR and EPA) and magnitudes (the average of 70 Mt CO_2_-eq yr^−1^ for EDGAR and 63 Mt CO_2_-eq yr^−1^ for EPA) over 1990–2021, different from those for PFC emissions submitted to UNFCCC (the average of 42 Mt CO_2_-eq yr^−1^ and the rate of − 2.6 Mt CO_2_-eq yr^−2^). UNFCCC reported the lowest PFC emissions before 2009 despite the same increasing trend. Moreover, the overall trend in PFC emissions after 2009 was decreasing in the UNFCCC reports but increasing in the EDAGR and EPA results. Figure 2e–h illustrates the overall higher emissions in EDGAR for each PFC than those from the UNFCCC (without individual PFC emissions provided in the EPA reports). C_2_F_6_ emission gaps between the EDGAR (23 Mt CO_2_-eq yr^−1^ in 1990 to 9.6 Mt CO_2_-eq yr^−1^ in 2021) and UNFCCC (17 Mt CO_2_-eq yr^−1^ in 1990 to 1.6 Mt CO_2_-eq yr^−1^ in 2021) were the highest, while C_3_F_8_ emission gaps between the EDGAR (0.21 Mt CO_2_-eq yr^−1^ in 1990 to 0.37 Mt CO_2_-eq yr^−1^ in 2021) and UNFCCC (0.22 Mt CO_2_-eq yr^−1^ in 1990 to 0.29 Mt CO_2_-eq yr^−1^ in 2021) were the lowest.

The discrepancies in inventory results may be brought by factors like emission source sector inclusion and country coverage in the inventories. Taking SF_6_ emission sources in the EDGAR and EPA as example, EDGAR covered four SF_6_ emission sources: chemical industry, metal industry, electronics industry, and other product manufacture and use; EPA covered electric power systems (EPS), electronics (manufacturing of semiconductors, photovoltaics and flat panel displays), and metal industry (magnesium production). EDGAR only provided general emission sector description like electronics industry and other product manufacture and use without detailed subsource, while EPA showed the subsource information of electronics industry. Besides, EPS, the major emission source of SF_6_ was not found in the EDGAR; the chemical industry was not contained in the EPA. Thus, it is hard to say which SF_6_ emission dataset has the most complete inputs and might therefore be most reliable. Combining multiple datasets makes it possible to obtain reliable emission estimates. In addition, the EPA and EDGAR both reported the NF_3_ emissions from non-Annex I countries. However, NF_3_ emissions from the non-Annex I countries were not available in the UNFCCC. With these missing data, it is not easy to determine whether there are no emissions or whether emissions were not calculated. This vague cognition would impair the accuracy of the existing BU estimates, which is not conducive to a correct understanding of the causes of the TD-BU differences. The above statements indicate that there is no consensus on the accounting of FFGHG emissions. It seems sectors and/or countries covered by previous inventories are different. Each inventory has its own disadvantages. Thus, more work such as identifying potential emission sources, including NF_3_ in national inventories of non-Annex I countries, and strengthening the national inventory reporting mechanism should be developed to further optimize existing BU results for FFGHGs in the future.

### Emission gap between TD and BU from a global perspective

Figure 2a shows the significant gap between global TD and BU total FFGHG emission estimates. Total FFGHG emissions here mean the sum of emissions of six individual FFGHG. First, albeit with fluctuations, global total FFGHG emissions from TD have shown an overall upward trend with an increasing rate of 5.3 Mt CO_2_-eq yr^−2^. However, three inventories showed diverse emission trends, partially different from the TD result. Among inventories, only the EDGAR inventory displayed a similar increase trend in global total FFGHG emissions but with an increase rate of 3.3 Mt CO_2_-eq yr^−2^. However, EPA’s report showed relatively steady FFGHG emissions ranging from 166 Mt CO_2_-eq yr^−1^ (1990) to 174 Mt CO_2_-eq yr^−1^ (2021). In addition, global total FFGHG emissions reported by the UNFCCC fluctuated widely and a decreasing trend could be found with a decreasing rate of 4.0 Mt CO_2_-eq yr^−2^ from 1990 to 2021. Noticeably, after 2009 global total FFGHG emissions from TD, the EDGAR inventory, and EPA’s report all showed an obvious increase with the rates of 11.6 Mt CO_2_-eq yr^−2^, 6.4 Mt CO_2_-eq yr^−2^, and 5.3 Mt CO_2_-eq yr^−2^, respectively. Figure 2a also shows a wide range in global total FFGHG emissions from TD and BU results. FFGHG emissions increased from 227 Mt CO_2_-eq yr^−1^ in 1983 to 424 Mt CO_2_-eq yr^−1^ in 2020 (purple squares), significantly higher than all BU results [UNFCCC: 149 Mt CO_2_-eq yr^−1^ (1990) to 25 Mt CO_2_-eq yr^−1^ (2020); EDGAR: 185 Mt CO_2_-eq yr^−1^ (1983) to 268 Mt CO_2_-eq yr^−1^ (2020); EPA: 166 Mt CO_2_-eq yr^−1^ (1990) to 167 Mt CO_2_-eq yr^−1^ (2020)]. The difference between TD estimates and UNFCCC BU estimates may result from emission underestimates of activity-based inventories as well as from substantial emissions from non-reporting countries. However, the causes for the differences between TD and EPA/EDGAR inventories are not fully known.

The emission gaps are also found in the individual FFGHG (Fig. 2b–h). From 1978 to 2020, the EDGAR inventory shows the average global SF_6_ emission of 124 Mt CO_2_-eq yr^−1^ and an increasing rate of 3.9 Mt CO_2_-eq yr^−2^, consistent with those from TD (the average emission of 134 Mt CO_2_-eq yr^−1^ and the increase rate of 3.9 Mt CO_2_-eq yr^−2^) (Fig. 2b). However, there are obvious discrepancies between the EDGAR inventory and TD results for both NF_3_ (Fig. 2c) and PFCs (Fig. 2d–h). Especially, the discrepancies between the EDGAR and TD results for NF_3_, CF_4_, C_2_F_6_, and *c*-C_4_F_8_ have gradually increased. Figure 2b–d illustrates that despite the similar trend, the EPA estimates for SF_6_ (63 Mt CO_2_-eq yr^−1^ in 1990 to 100 Mt CO_2_-eq yr^−1^ in 2020), NF_3_ (0.24 Mt CO_2_-eq yr^−1^ in 1990 to 9.9 Mt CO_2_-eq yr^−1^ in 2020), and PFCs (103 Mt CO_2_-eq yr^−1^ in 1990 to 57 Mt CO_2_-eq yr^−1^ in 2020) were significantly lower than the global TD emissions for SF_6_ (118 Mt CO_2_-eq yr^−1^ in 1990 to 205 Mt CO_2_-eq yr^−1^ in 2020), NF_3_ (0.6 Mt CO_2_-eq yr^−1^ in 1990 to 68 Mt CO_2_-eq yr^−1^ in 2020), and PFCs (157 Mt CO_2_-eq yr^−1^ in 1990 to 167 Mt CO_2_-eq yr^−1^ in 2020), respectively. In addition, UNFCCC estimates for each FFGHG were also lower than those from global TD results. Figure 2 displays the gradual decline in global emissions for SF_6_, CF_4_, and C_2_F_6_ reported by the UNFCCC with the decreasing rate of 1.4, 2.1, and 0.50 Mt CO_2_-eq yr^−2^, as well as the gradual increasing discrepancies between the UNFCCC and TD results for SF_6_, CF_4_, and C_2_F_6_. It is also worth paying attention to the significant emission gaps for *c*-C_4_F_8_ between the average global TD (12.8 Mt CO_2_-eq yr^−1^ over 1990–2020) and UNFCCC (0.28 Mt CO_2_-eq yr^−1^ over 1990–2020).

### Emission gap among TD from a regional perspective

The regional TD emission estimation is usually carried out based on the location of existing atmospheric observation stations. Previous regional TD studies for FFGHGs have been gathered in Supplementary Table 3, indicating that existing TD research on FFGHG emissions mainly focused on the following regions: eastern Asia (China; Japan; South Korea; North Korea; and Mongolia), northwest Europe (referring to terms “northwestern Europe/West Europe/northwest Europe” used in previous studies) [Austria; Belgium, the Netherlands, and Luxembourg (collectively termed Benelux); Denmark; France; Germany; Ireland; Italy; Portugal; Spain; Switzerland; and the United Kingdom (UK)], the US, Australia, India, and Russia.

**Figure 3:**
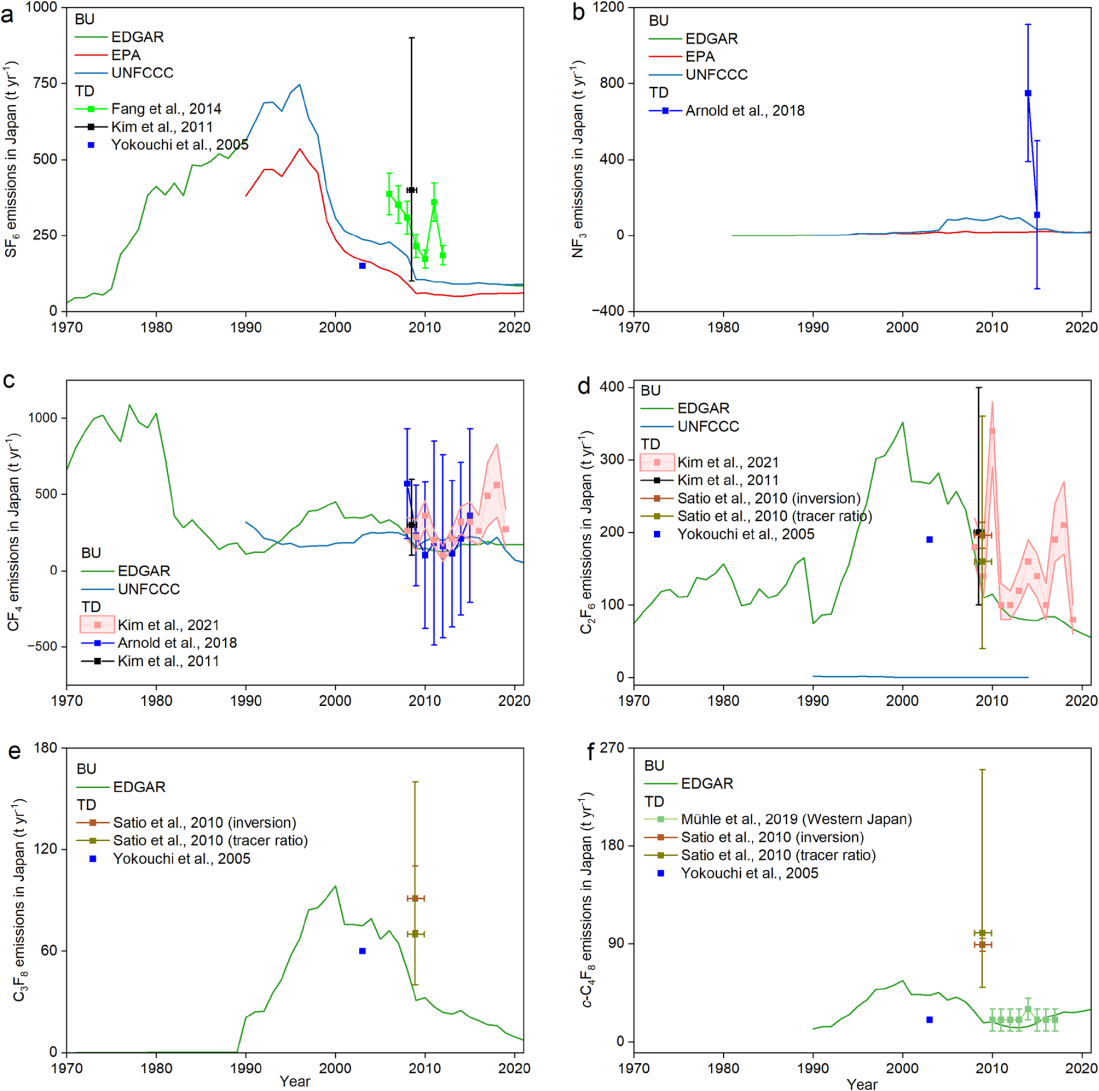
Summary of TD and BU FFGHG emissions in Japan from previous studies for (**a**) SF_6_, (**b**) NF_3_, (**c**) CF_4_, (**d**) C_2_F_6_, (**e**) C_3_F_8_, and (**f**) *c*-C_4_F_8_. Unit: tons per year (t yr^−1^). The detailed sources can be found in Supplementary Table 3. FFGHG emissions in the United Nations Framework Convention on Climate Change (UNFCCC) are obtained from the following website: https://di.unfccc.int/flex_annex1 and https://di.unfccc.int/flex_non_annex1. FFGHG emissions in the Emissions Database for Global Atmospheric Research (EDGAR) are from EDGAR v4.2^37^ (1970–1989) and EDGAR v7.0^38^ (1990–2021). FFGHG CO_2_-equivalent emissions in US Environmental Protection Agency (EPA) were from Global Non-CO_2_ Greenhouse Gas Emission Projections & Marginal Abatement Cost Analysis: Methodology Documentation^39^. Note that EPA only provided total PFC emissions instead of individual PFC emissions. All TD and BU data is accessed before 2023-11-10.

The FFGHG TD estimates from the above regions except for China (provided by Guo et al., 20023^27^) are shown in Fig. 3 and Supplementary Figs. 1–10. Among these estimates, only one TD result for specific FFGHG is available in regions and countries including China (NF_3_), Japan (NF_3_), South Korea (NF_3_), North Korea (SF_6_/NF_3_/CF_4_/C_2_F_6_/C_3_F_8_), Mongolia (SF_6_), northwest Europe (CF_4_/C_2_F_6_/C_3_F_8_/*c*-C_4_F_8_), and the US (SF_6_). These limited TD results are not sufficient to understand FFGHG emissions from these regions. Thus, more work on the emission quantification of FFGHGs in these regions by the TD method should be developed to further verify the previous TD results. Note that for China (SF_6_/CF_4_/C_2_F_6_/C_3_F_8_/*c*-C_4_F_8_), Japan (SF_6_/CF_4_/C_2_F_6_/C_3_F_8_/*c*-C_4_F_8_), South Korea (SF_6_/CF_4_/C_2_F_6_/C_3_F_8_/*c*-C_4_F_8_), North Korea (*c*-C_4_F_8_), northwest Europe (SF_6_), and Australia (SF_6_/CF_4_/C_2_F_6_/C_3_F_8_/*c*-C_4_F_8_), more than one TD results for each FFGHG are accessible. If considering the emission uncertainties, parts of regional TD FFGHG emissions were relatively consistent. For example, Fig. 3 shows that FFGHG emissions in Japan from different TD studies were close to each other. Supplementary Fig. 1 shows the consistency among different TD studies for SF_6_/CF_4_/C_2_F_6_/C_3_F_8_ emissions in South Korea.

Supplementary Figure 4 displayed the relatively close four groups of SF_6_ emissions in northwest Europe shown by Simmonds et al., 2020^1^ despite using different inverse models and observation data with different number of sampling points and sampling years. However, there are gaps among part of the regional TD results. For example, Supplementary Fig. 10a shows that Australian SF_6_ emissions using the interspecies correlation (ISC) method (68 ± 25 t yr^−1^ in 2005 to 18 ± 6 t yr^−1^ in 2016)^28^ were quite different from those using the InTEM model (29 ± 2 t yr^−1^ in 2005 to 44 ± 2 t yr^−1^ in 2016)^28^. Similarly, Supplementary Fig. 10d shows the differences between the Australian C_3_F_8_ emissions using the ISC method (7 ± 3 t yr^−1^ in 2005 to 9 ± 3 t yr^−1^ in 2016)^28^ and those using the NAME model (9 ± 1 t yr^−1^ in 2005 to 20 ± 2 t yr^−1^ in 2016)^28^. This means that the selection of TD method covering inversion model, prior emissions, observations, and uncertainties would impact the TD result. In addition, Guo et al., 2023^27^ also show obvious discrepancies among TD emissions for SF_6_/CF_4_/C_2_F_6_/*c*-C_4_F_8_ in China.

For previous TD studies, the lack of atmospheric measurement data from existing stations would impede the accurate understanding of long-term FFGHG emissions. For example, Say et al., 2021^19^ only reported emissions (2005–2010 for CF_4_; 2005–2007 for C_2_F_6_ and C_3_F_8_) from the UK, Ireland, and Benelux due to the lack of atmospheric measurements during this period from continental Europe and thus sensitivity to southern France and eastern Germany. In addition, due to the availability of measurements from Jungfraujoch station, reported estimates for France and Germany (and Northwest Europe total) began in 2008 (C_2_F_6_ and C_3_F_8_) and 2010 (CF_4_)^19^.

Besides, the lack of atmospheric measurement stations would not be conducive to an accurate understanding of FFGHG emissions. For example, Mühle et al., 2019^24^ indicated that several large areas such as the US and India where *c*-C_4_F_8_ emissions may occur were not closely monitored by the AGAGE network. *c*-C_4_F_8_ emissions from the continental US were not estimated because two AGAGE stations in California could only catch part of the *c*-C_4_F_8_ emissions from the continental US due to predominant westerly winds^24^. For India, the inversion method played a limited role in identifying distant point sources from a relatively small number of samples^24^. Weiss et al., 2021^29^ pointed out that vast blind spots exist in the AGAGE and NOAA measurement networks which include large parts of the developed regions relatively well sampled such as eastern Asia, central North America, and northwest Europe as summarized in our work; however, southern, western, and central Asia, large parts of Southeast Asia, all of South America, portions of North America, Eastern Europe, and New Zealand and most of Africa are not covered and emissions from many of these areas are expected to increase with industrial and economic development^29^.

Overall, more TD research on regional FFGHG emission quantification needs to be carried out to verify the previous results and reduce the uncertainties of FFGHG emissions. Besides, atmospheric measurements from the current regional atmospheric observation should be further completed. More atmospheric observation stations should be developed as well to expand coverage of potential emission areas and then improve the accuracy of atmospheric measurements.

### Emission gap among BU from a regional perspective

**Figure 4:**
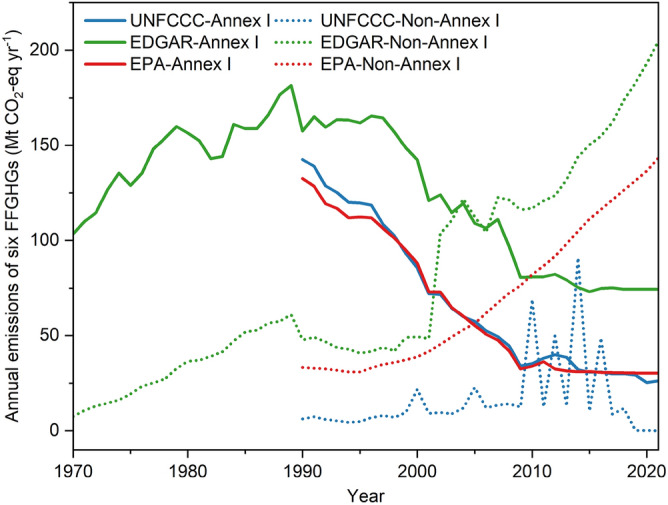
FFGHG CO_2_-equivalent emissions for Annex I countries and non-Annex I countries derived from several inventories from 1970 to 2021. Unit: million tons CO_2_-equivalent per year (Mt CO_2_-eq yr^−1^). Historical emission estimates of individual FFGHG species in UNFCCC for Annex I countries and non-Annex I countries were from the UNFCCC flexible query for Annex I (https://di.unfccc.int/flex_annex1) and the UNFCCC flexible query for non-Annex I (https://di.unfccc.int/flex_non_annex1), respectively. However, NF_3_ emissions for non-Annex I countries were not available on the UNFCCC website. FFGHG emissions in the Emissions Database for Global Atmospheric Research (EDGAR) are from EDGAR v4.2^37^ (1970–1989) and EDGAR v7.0^38^ (1990–2021). FFGHG CO_2_-equivalent emissions in US Environmental Protection Agency (EPA) were from Global Non-CO_2_ Greenhouse Gas Emission Projections & Marginal Abatement Cost Analysis: Methodology Documentation^39^. All data is accessed before 2023-11-10.

Figure 4 compares the FFGHG emissions between Annex I countries and non-Annex I countries from three inventories. Note that the EDGAR inventory reported the highest FFGHG emissions for both Annex I countries and non-Annex I countries.

For Annex I countries, three inventories all showed similar decline trends in FFGHG emissions at a decreasing rate of 2.7–3.8 Mt CO_2_-eq yr^−2^, indicating the long-term efforts of these countries in reducing FFGHG emissions in industries like electrical equipment, and semiconductor manufacturing^30,31^. Among three inventories, FFGHG emissions from Annex I countries reported by the UNFCCC (143 Mt CO_2_-eq yr^−1^ in 1990 to 26 Mt CO_2_-eq yr^−1^ in 2021) are consistent with those shown by the EPA inventory (133 Mt CO_2_-eq yr^−1^ in 1990 to 30 Mt CO_2_-eq yr^−1^ in 2021), but substantially lower than the results from EDGAR inventory (158 Mt CO_2_-eq yr^−1^ in 1990 to 75 Mt CO_2_-eq yr^−1^ in 2021). For non-Annex I countries, EPA and EDGAR inventories show an increasing trend in historical FFGHG emissions (red and green short-dot lines, respectively). Moreover, these two inventories indicate that FFGHG emissions from non-Annex I countries have surpassed those from Annex I countries since 2005. However, the FFGHG emissions from non-Annex I countries (blue short-dot lines) shown by the UNFCCC were relatively stable except for several peaks (2000, 2005, 2010, 2012, 2014, 2016). According to the limited FFGHG emission data for non-Annex I countries (Supplementary Fig. 11) from UNFCCC, these emission peaks were mainly caused by FFGHG emissions from China, India, and South Korea. The increasing trend in FFGHG emissions from the above three countries can also be found in Supplementary Fig. 11. FFGHG emissions from non-Annex I countries (mainly developing countries) do not have to be reported to the UNFCCC, indicating the possible missing of emission data from non-Annex I countries and thus causing the emission trend (blue short-dot lines) in Fig. 4. To narrow the emission gaps among inventories for non-Annex I countries, it is necessary to further improve the FFGHG inventories reported to the UNFCCC for non-Annex I countries.

Figure 3 and Supplementary Figs. 1–10 also show comparisons of FFGHG BU emissions for different regions and countries [without BU emissions available for North Korea (CF_4_/C_2_F_6_/C_3_F_8_/*c*-C_4_F_8_) and Australia (*c*-C_4_F_8_)]. For example, for SF_6_, NF_3_, and CF_4_, the BU emissions in Japan were consistent, while the C_2_F_6_ emissions reported by EDAGR (75 t yr^−1^ in 1990 to 56 t yr^−1^ in 2021) were higher than those from UNFCCC (2.1 t yr^−1^ in 1990 to 0.59 t yr^−1^ in 2021). For South Korea, only the BU SF_6_ emissions from 1990 to 2014 were close to each other. EPA and UNFCCC reported higher NF_3_ and PFCs emissions in South Korea than EDGAR, respectively. Supplementary Fig. 4 shows that SF_6_ emissions in West Europe submitted to UNFCCC (478 t yr^−1^ in 1990 to 209 t yr^−1^ in 2021) were close to the results from EDGAR (375 t yr^−1^ in 1990 to 296 t yr^−1^ in 2021) but higher than those from EPA (214 t yr^−1^ in 1990 to 76 t yr^−1^ in 2021). Supplementary Figs. 5–8 indicate that CF_4_/C_2_F_6_/C_3_F_8_/*c*-C_4_F_8_ emissions in Northwest Europe from EDGAR and UNFCCC were getting closer to each other, especially in the most recent ten years. For the US, the SF_6_ emissions from UNFCCC (1.3 t yr^−1^ in 1990 to 0.34 t yr^−1^ in 2021) were close to the results from EPA (1.3 t yr^−1^ in 1990 to 0.22 t yr^−1^ in 2021) but lower than those from EDGAR (2.1 t yr^−1^ in 1990 to 1.3 t yr^−1^ in 2021) (Supplementary Fig. 9). Hu et al.^2^ indicated that the US SF_6_ emissions from EDGAR were up to 5 times larger than the emissions in their reporting to the UNFCCC largely because of the electric power transmission and distribution (ETD) sector. For the SF_6_/CF_4_/C_2_F_6_ emissions in Australia (Supplementary Fig. 10), there are similar emission trends and magnitudes among previous inventories.

### Emission gap between TD and BU from a regional perspective

Combining the accessible regional FFGHG emissions from TD studies and emission inventories (EDGAR, EPA, and UNFCCC), the comparison of TD and BU FFGHG emission estimates for Japan, South Korea, North Korea, Mongolia, northwest Europe, the US, and Australia have been shown in Fig. 3 and Supplementary Figs. 1–10 and described in the following. Note that no comparison was made for North Korea (CF_4_/C_2_F_6_/C_3_F_8_/*c*-C_4_F_8_) and Australia (*c*-C_4_F_8_) because of the lack of BU emissions.

For Japan, the TD SF_6_ emission reported by Kim et al., 2011^32^ [400 (100–900) t yr^−1^ over 2007–2008], the TD NF_3_ emission (110 ± 390 t yr^−1^ in 2015), TD CF_4_ emissions (average value ranging from 90 to 250 t yr^−1^ over 2008–2015), TD C_2_F_6_ emissions (average value of 184 t yr^−1^ in 2008), and TD *c*-C_4_F_8_ emissions (21 t yr^−1^ over 2010–2017) were close to the corresponding BU results [SF_6_: 119–206 t yr^−1^ in 2007 & 91–182 t yr^−1^ in 2008; NF_3_: 22–33 t yr^−1^ in 2015; CF_4_: 155–205 t yr^−1^ over 2008–2015; C_2_F_6_: 0.33–174 t yr^−1^ in 2008; *c*-C_4_F_8_: 17 t yr^−1^ over 2010–2017]. However, there are obvious differences between the average TD and BU results for SF_6_ emissions during 2006–2012 (TD: 233–332 t yr^−1^; BU: 82–146 t yr^−1^) from Fang et al., 2014^33^, NF_3_ emission in 2014 (TD: 750 ± 332 t yr^−1^; BU: 20–65 t yr^−1^), CF_4_ emissions during 2017–2019 (TD: 263–647 t yr^−1^; BU: 175 t yr^−1^), C_2_F_6_ emissions in 2010 & during 2013–2018 (TD: 290–380 t yr^−1^ & 125–192 t yr^−1^; BU: 0.23–115 t yr^−1^ & 0.088–81 t yr^−1^), C_3_F_8_ emissions in 2009 (TD: 40–160 t yr^−1^; BU: 31 t yr^−1^), and *c*-C_4_F_8_ emissions in 2009 (TD: 50–150 t yr^−1^ BU: 18 t yr^−1^). In addition, the FFGHG emissions reported by Yokouchi et al., 2005^34^ were all lower than those from EDGAR (Fig. 3). In Supplementary Fig. 1, the TD CF_4_/C_2_F_6_/C_3_F_8_ emissions in South Korea^4,7,32,35^ were close to the average UNFCCC results but higher than those from EDGAR. SF_6_ emissions reported by Fang et al., 2014^33^ (374–640 t yr^−1^ over 2006–2012), NF_3_ emissions reported by Arnold et al., 2018^7^ (400–560 t yr^−1^ over 2014–2015), and *c*-C_4_F_8_ emissions reported by Satio et al., 2010^35^ (32 t yr^−1^ over 2007–2009) in South Korea were also higher than the BU results (SF_6_: 295–340 t yr^−1^ over 2006–2012; NF_3_: 105 t yr^−1^ over 2014–2015; *c*-C_4_F_8_: 0.0093–10 t yr^−1^ over 2007–2009). For the TD SF_6_ and NF_3_ emissions in North Korea (− 2.8–101 t yr^−1^ of SF_6_ during 2006–2012 and − 55–255 t yr^−1^ of NF_3_ during 2014–2015) (Supplementary Fig. 2) and TD SF_6_ emissions in Mongolia [− 22 to 76 t yr^−1^ during 2006–2012] (Supplementary Fig. 3), the average results were relatively close to the average BU results (North Korea: 5.0 t yr^−1^ of SF_6_ during 2006–2012; North Korea: 0 t yr^−1^ of NF_3_ during 2014–2015; Mongolia: 1.0 t yr^−1^ of SF_6_ during 2006–2012) considering the large uncertainties of TD results.

Supplementary Fig. 4 displayed that SF_6_ emissions in northwest Europe from Simmonds et al., 2020^1^ were relatively close to the EDGAR and UNFCCC results but higher than those in EPA. Supplementary Figs. 5–7 show the consistency between the TD^19^ and BU emissions for CF_4_, C_2_F_6_, and C_3_F_8_ in Northwest Europe, respectively. However, the TD *c*-C_4_F_8_ emissions in northwest Europe reported by Mühle et al., 2019^24^ (26 ± 13 t yr^−1^ during 2013–2017) were higher than those from EDGAR (1.7 t yr^−1^ in 2013 to 1.3 t yr^−1^ in 2017) and UNFCCC (0.71 t yr^−1^ in 2013 to 0.72 t yr^−1^ in 2017) (Supplementary Fig. 8). Using NOAA’s ground-based and airborne measurements of SF_6_ to estimate SF_6_ emissions from the United States, Hu et al., 2023^2^ reported TD SF_6_ emissions in the US (0.83 ± 0.19 t yr^−1^ in 2007 to 0.39 ± 0.12 t yr^−1^ in 2018) were higher than the results from EPA (0.40 t yr^−1^ in 2007 to 0.25 t yr^−1^ in 2018) and UNFCCC (0.41 t yr^−1^ in 2007 to 0.25 t yr^−1^ in 2018) but lower than those from EDGAR (1.5 t yr^−1^ in 2007 to 1.3 t yr^−1^ in 2018) (Supplementary Fig. 9). In Supplementary Fig. 10, the TD CF_4_ emissions^28,36^ in Australia were close to those from EDGAR and UNFCCC. However, reported TD SF_6_ and C_3_F_8_ emissions were higher than the BU results in Australia. For C_2_F_6_, only the emissions in Australia reported by Dunse et al., 2018 using the TAPM/NAME average^28^ were close to the BU results. Overall, there is a consensus with the TD/BU performance for CF_4_ in Japan, SF_6_&NF_3_ in North Korea, C_2_F_6_&C_3_F_8_ in South Korea, SF_6_ in Mongolia, CF_4_&C_2_F_6_&C_3_F_8_ in northwest Europe, and CF_4_ in Australia, if considering the uncertainties of FFGHG emissions.

### Implications of FFGHG emissions

At present, the world is actively coping with climate change. Actively promoting FFGHG reduction is conducive to addressing climate change. Emission reduction of FFGHGs is based on the accurate understanding of FFGHG emissions. However, previous emission results from atmospheric observation and inventories are not accurate enough to assist with the FFGHG mitigation. Combined with the latest emission data, this work analyzed the emission gaps from global and regional perspectives, and the obvious emission gaps were found among different regional TD results, between the TD and BU results from the global and regional perspectives, as well as among different inventories at the global and regional scales. These emission gaps revealed certain problems. First, the existing emission inventories could not match each other well. Second, the regional TD studies were still limited, especially for China (NF_3_), Japan (NF_3_), South Korea (NF_3_), North Korea (SF_6_/NF_3_/CF_4_/C_2_F_6_/C_3_F_8_), Mongolia (SF_6_), Northwest Europe (CF_4_/C_2_F_6_/C_3_F_8_/*c*-C_4_F_8_), and the US (SF_6_). Third, the FFGHG emissions in certain areas were not estimated due to the lack of atmospheric observation data/stations, especially in areas with potential FFGHG emissions. Finally, BU inventories could miss parts of potential FFGHG emissions, especially for non-Annex I countries, also possibly underestimating the FFGHG emissions. Thus, based on the above problems, future research should focus on the following aspects to establish a support system to provide scientific support for FFGHG mitigation: (1) further improving the accuracy of current inventories to verify each other well; (2) further completing atmospheric measurement data from the current atmospheric observation by optimizing the data monitoring process; (3) conducting more regional TD studies to verify previous TD FFGHG emissions and thus obtaining more accurate regional TD FFGHG emissions; (4) establishing more atmospheric observation stations to cover as many potential FFGHG emission areas as possible; (5) helping the completion of the emission inventories of non-Annex I countries. Through the above movements, it is expected to further understand the precise emissions of FFGHGs in the future to support the FFGHG emission reduction and thus climate change mitigation.

## Methods

In this work, TD and BU FFGHG emissions at the global and regional scales are collected first. The global and regional TD emissions for SF_6_, NF_3_, CF_4_, C_2_F_6_, C_3_F_8_, and *c*-C_4_F_8_ are collected from previous studies. The global TD total FFGHG emissions are calculated as the sum of emissions of six FFGHGs. The national BU emissions for SF_6_, NF_3_, CF_4_, C_2_F_6_, C_3_F_8_, and *c*-C_4_F_8_ are collected from the EDGAR, EPA, and UNFCCC. The global BU total FFGHG emissions from the EDGAR, EPA, and UNFCCC are calculated as the sum of emissions of six FFGHGs from all countries.

Based on the above data, we explore and analyze the FFGHG emission gaps by comparing TD and BU results at the global and regional levels from the following six aspects: (1) emission gap among TD at the global level; (2) emission gap among inventories at the global level; (3) emission gap between TD and BU at the global level; (4) emission gap among TD at the regional level; (5) emission gap among inventories at the regional level; (6) emission gap between TD and BU at the regional level. The gaps are reflected in the following two aspects: (a) differences in emission trend; (b) differences in emission magnitude.

A Monte Carlo (MC) ensemble simulation was performed to calculate the uncertainties in the global total PFC emissions and the global total six FFGHG emissions. The emission model was run 1,000,000 times by randomly varying all the input data given a priori uncertainty distributions. The normal distribution was applied for all emission data.

### Supplementary Information

Supplementary Information.

## Data Availability

The top-down annual global FFGHG emissions were the sum of six FFGHG global emissions from Simmonds et al.^1^, Say et al.^19^, Mühle et al.^23,24^, updates of Rigby et al.^16^, and WMO, 2022^18^. FFGHG emissions in the United Nations Framework Convention on Climate Change (UNFCCC) are obtained from the following website: https://di.unfccc.int/flex_annex1 and https://di.unfccc.int/flex_non_annex1. FFGHG emissions in the Emissions Database for Global Atmospheric Research (EDGAR) are from EDGAR v4.2^37^ (1970–1989) and EDGAR v7.0^38^ (1990–2021). FFGHG CO_2_-equivalent emissions in U.S. Environmental Protection Agency (EPA) were from Global Non-CO_2_ Greenhouse Gas Emission Projections & Marginal Abatement Cost Analysis: Methodology Documentation^39^. All data is accessed before 2023-11-10.
