# Supplementary material for: Revealing the global emission gaps for fully fluorinated greenhouse gases
Source: Sci Rep. 2024 Apr 16;14:8753. doi: 10.1038/s41598-024-58504-x (PMC11021409; doi:10.1038/s41598-024-58504-x)
Supplement: Supplementary file 1 — Supplementary Information. [file 41598_2024_58504_MOESM1_ESM.docx]

Revealing the global emission gaps for fully fluorinated greenhouse gases

Liya Guo^1^, Xuekun Fang^1,2,*^

^1^College of Environmental & Resource Sciences, Zhejiang University, Hangzhou, Zhejiang 310058, China

^2^Center for Global Change Science, Massachusetts Institute of Technology, Cambridge, MA 02139, United States

**Supplementary Figures**


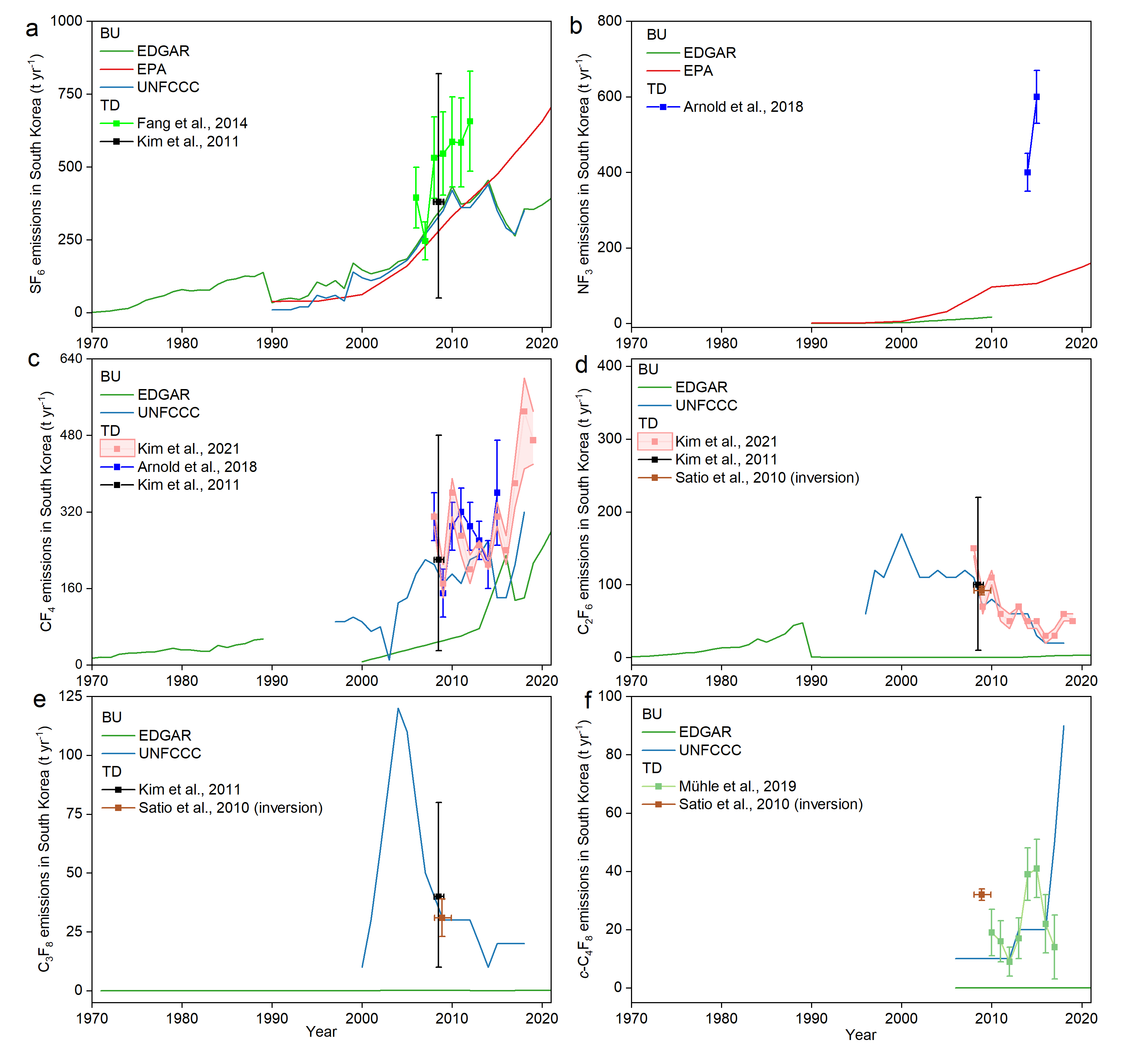


**Supplementary Figure 1**. Summary of TD and BU FFGHG emissions in South Korea from previous studies for (**a**) SF_6_, (**b**) NF_3_, (**c**) CF_4_, (**d**) C_2_F_6_, (**e**) C_3_F_8_, and (**f**) *c*-C_4_F_8_.The detailed sources can be found in Supplementary Table 3.


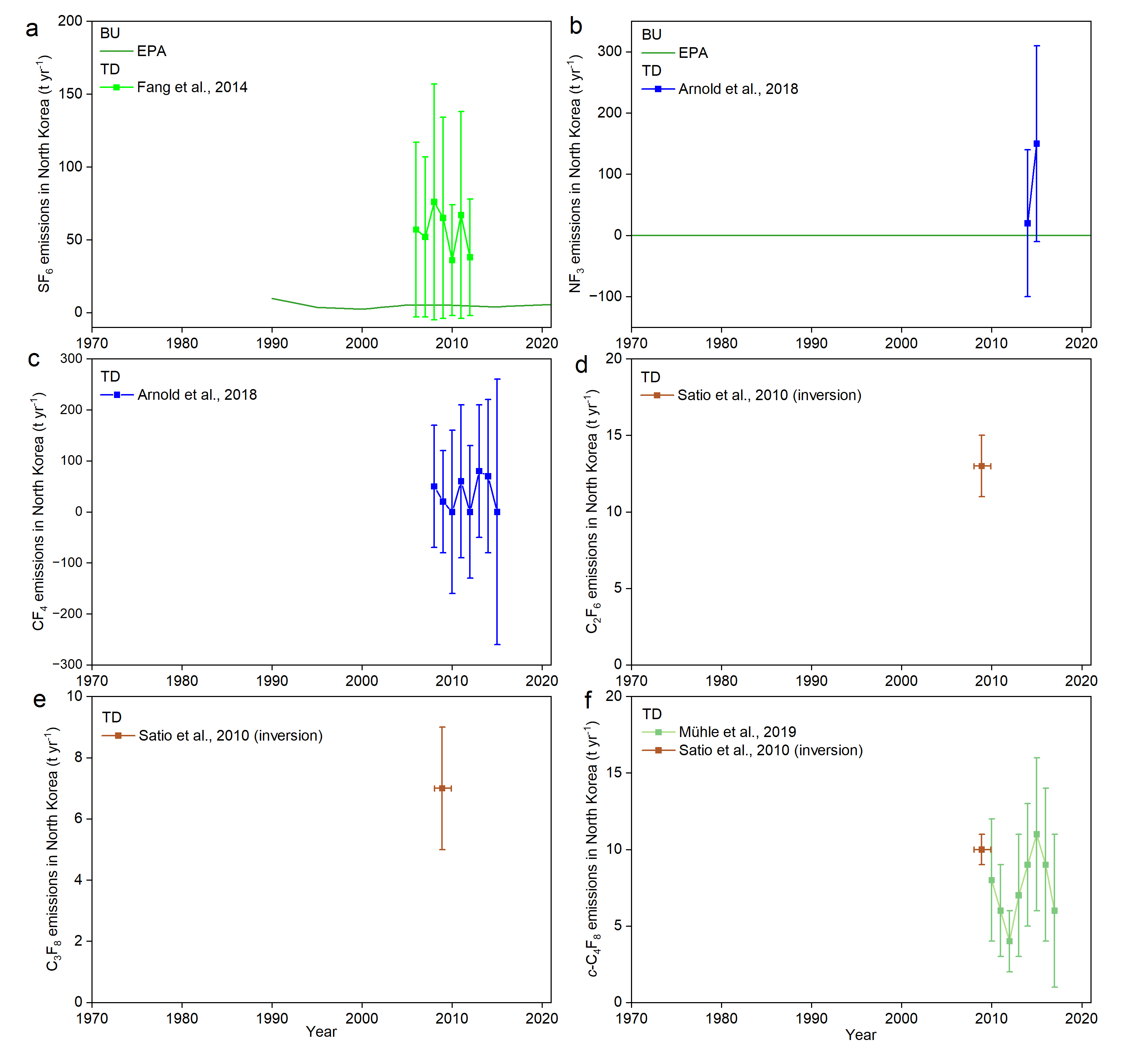


**Supplementary Figure 2**. Summary of TD and BU FFGHG emissions in North Korea from previous studies for (**a**) SF_6_, (**b**) NF_3_, (**c**) CF_4_, (**d**) C_2_F_6_, (**e**) C_3_F_8_, and (**f**) *c*-C_4_F_8_. The detailed sources can be found in Supplementary Table 3.


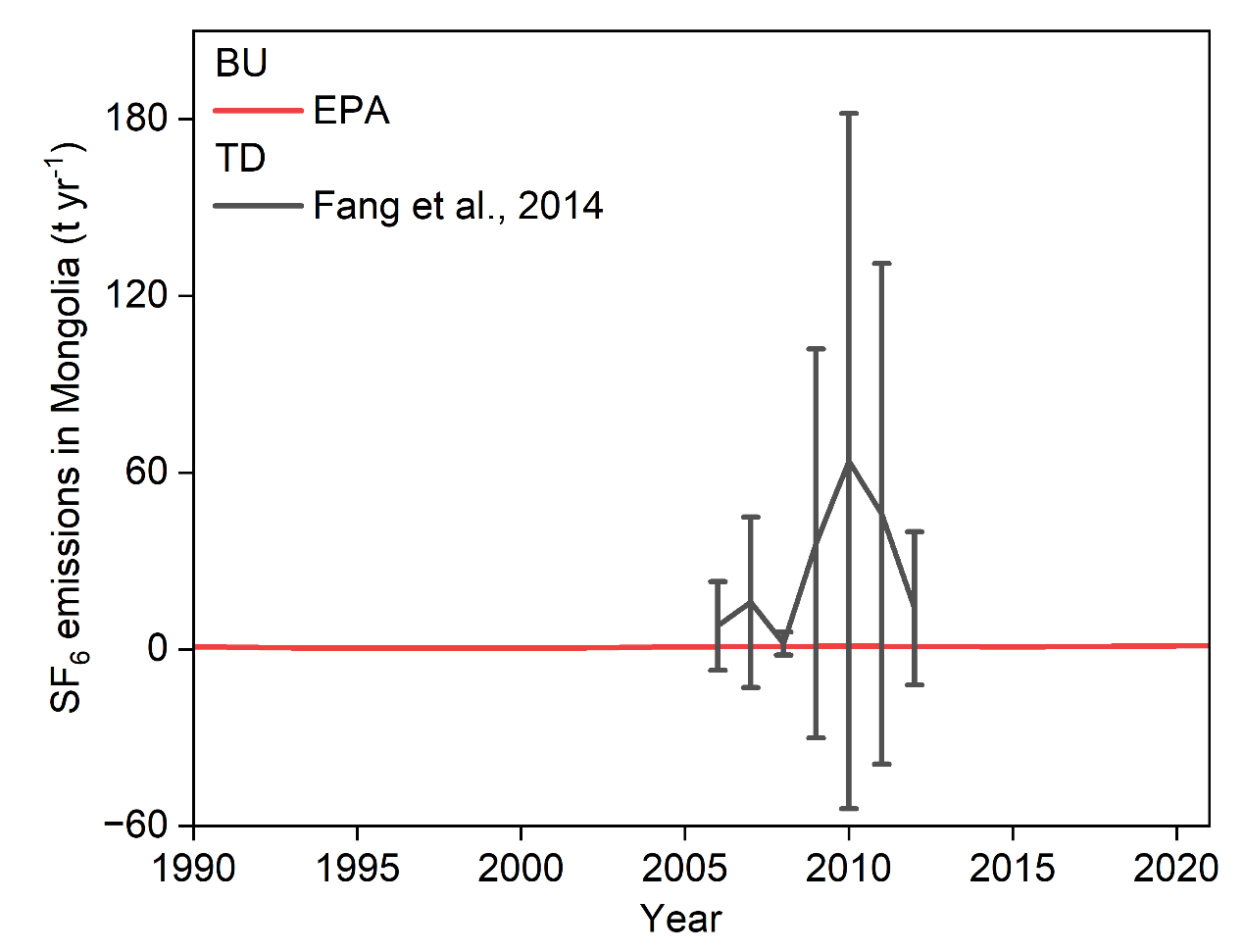


**Supplementary Figure 3**. Summary of TD and BU SF_6_ emissions in Mongolia from previous studies. The detailed sources can be found in Supplementary Table 3.


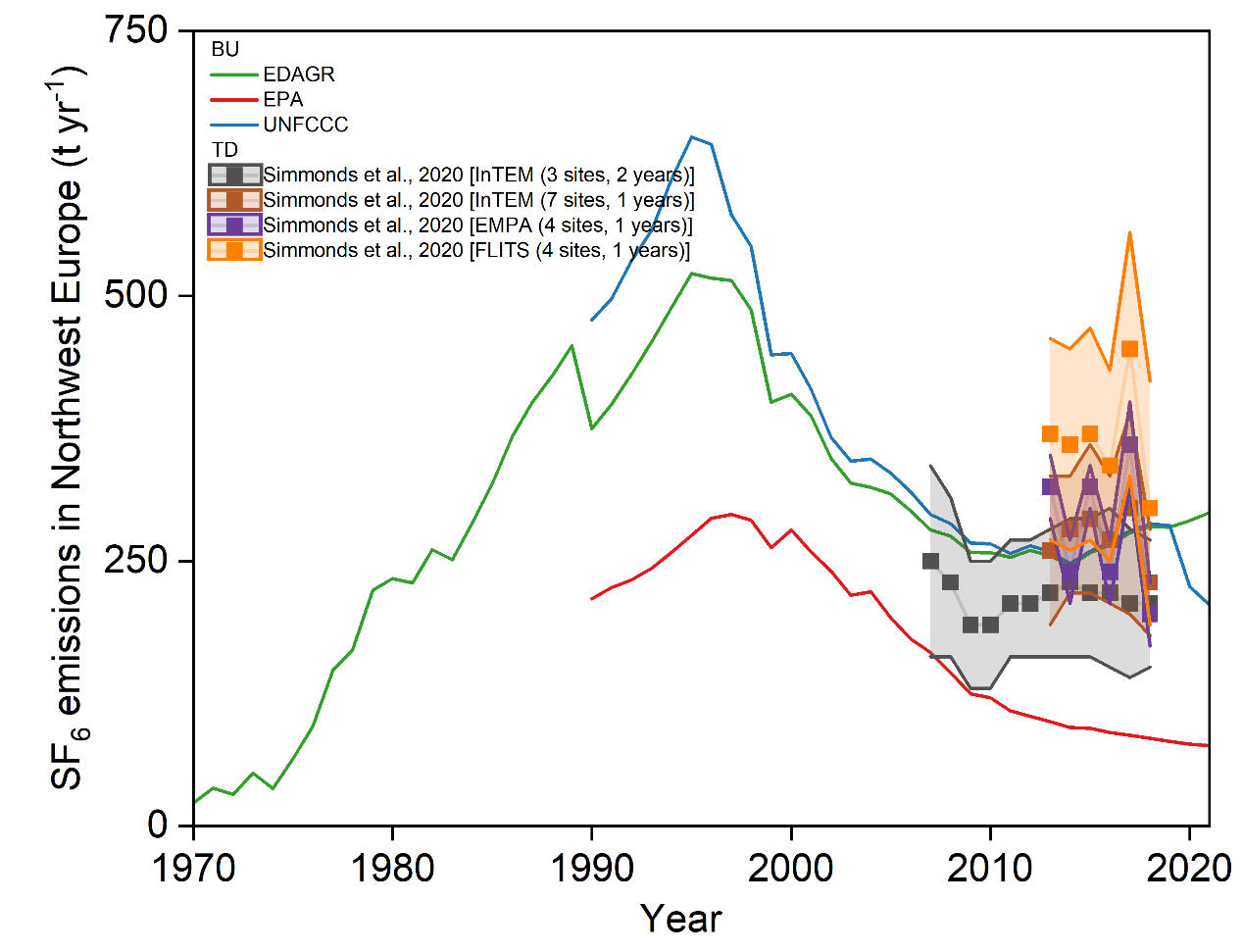


**Supplementary Figure 4**. Summary of TD and BU SF_6_ emissions in Northwest Europe from previous studies. The detailed sources can be found in Supplementary Table 3.


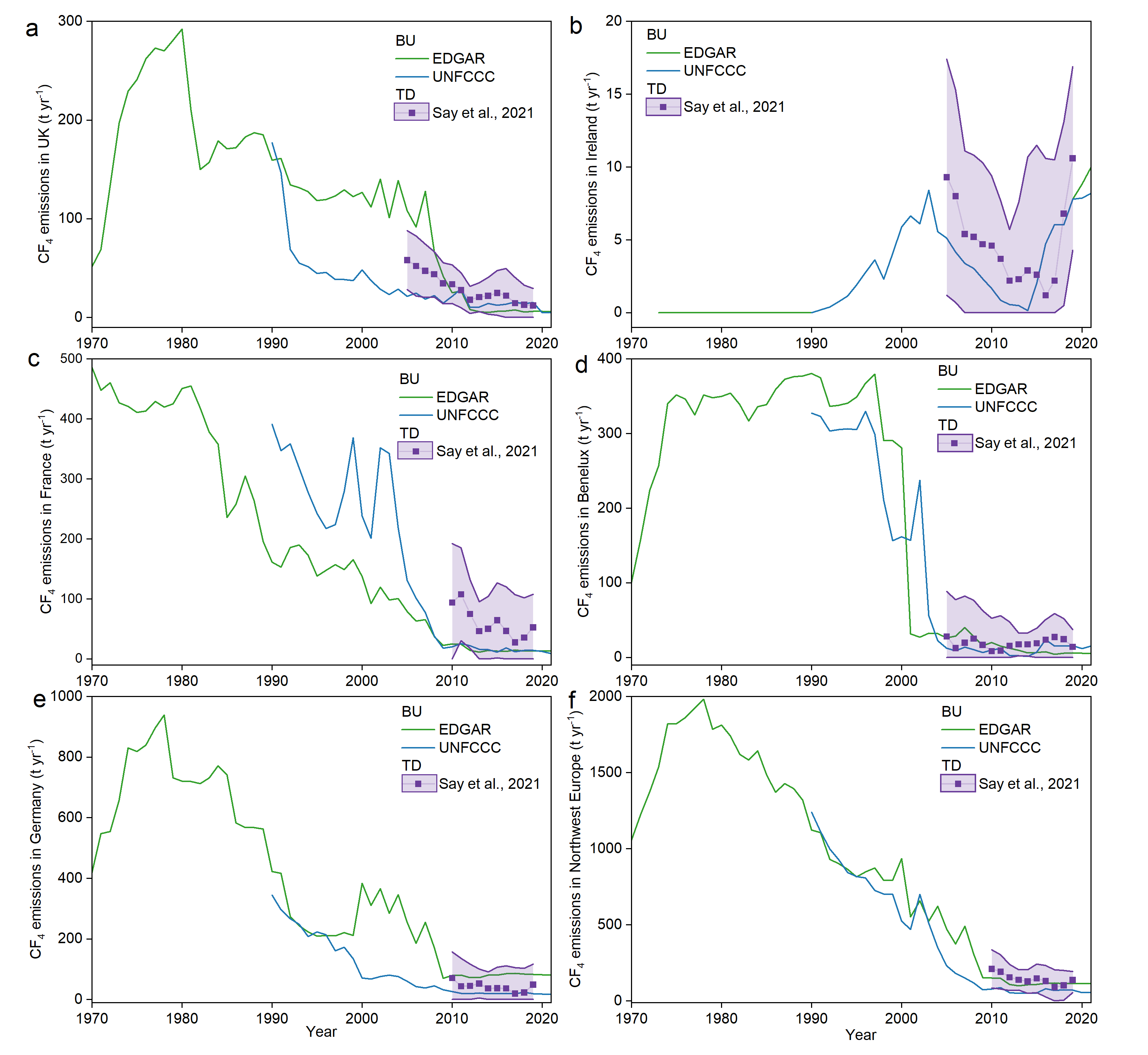


**Supplementary Figure 5**. Summary of TD and BU CF_4_ emissions in Northwest Europe from previous studies for (**a**) UK, (**b**) Ireland, (**c**) France, (**d**) Benelux, (**e**) Germany, and (**f**) total Northwest Europe. The detailed sources can be found in Supplementary Table 3.


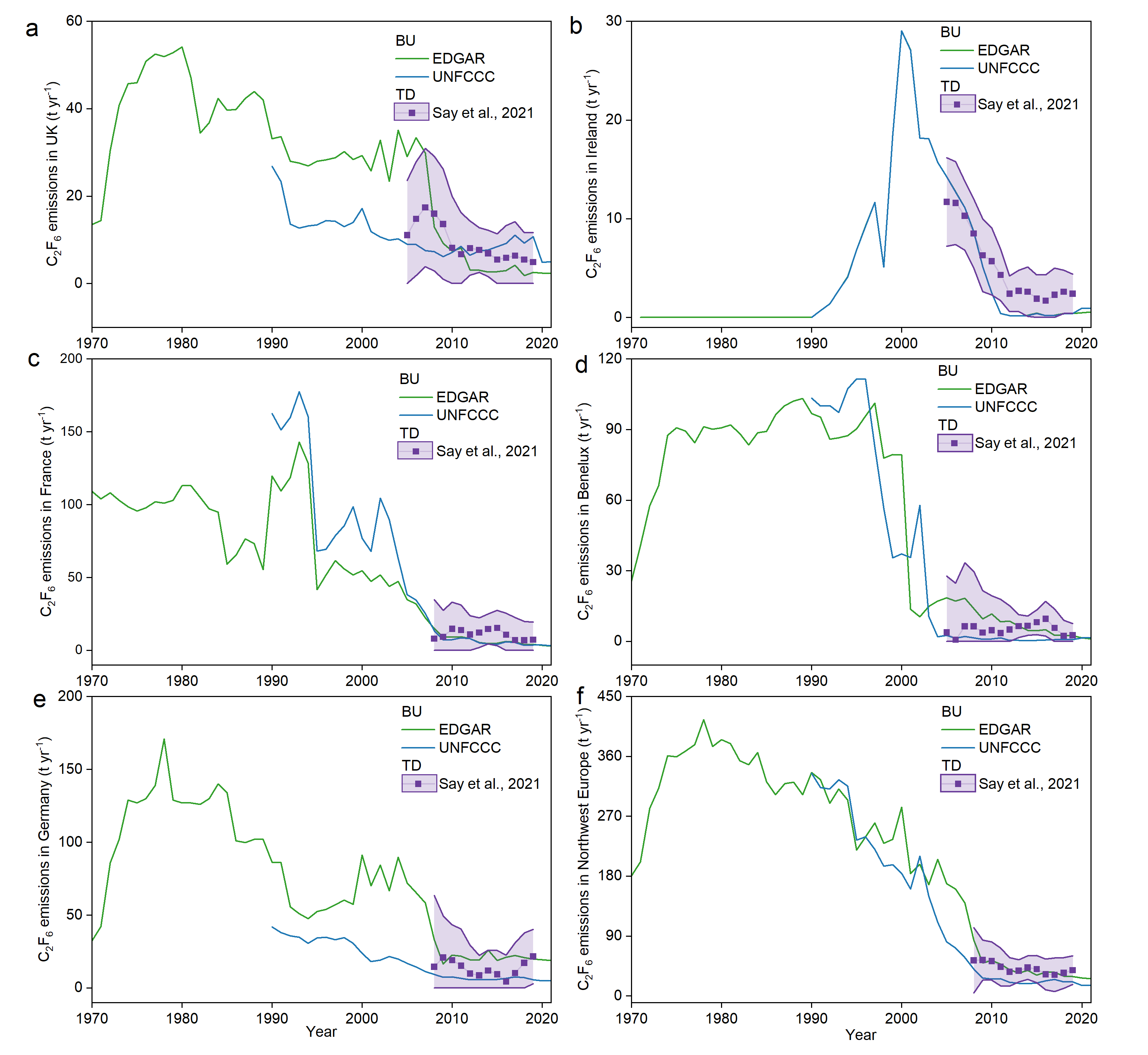


**Supplementary Figure 6**. Summary of TD and BU C_2_F_6_ emissions in Northwest Europe from previous studies for (**a**) UK, (**b**) Ireland, (**c**) France, (**d**) Benelux, (**e**) Germany, and (**f**) total Northwest Europe. The detailed sources can be found in Supplementary Table 3.


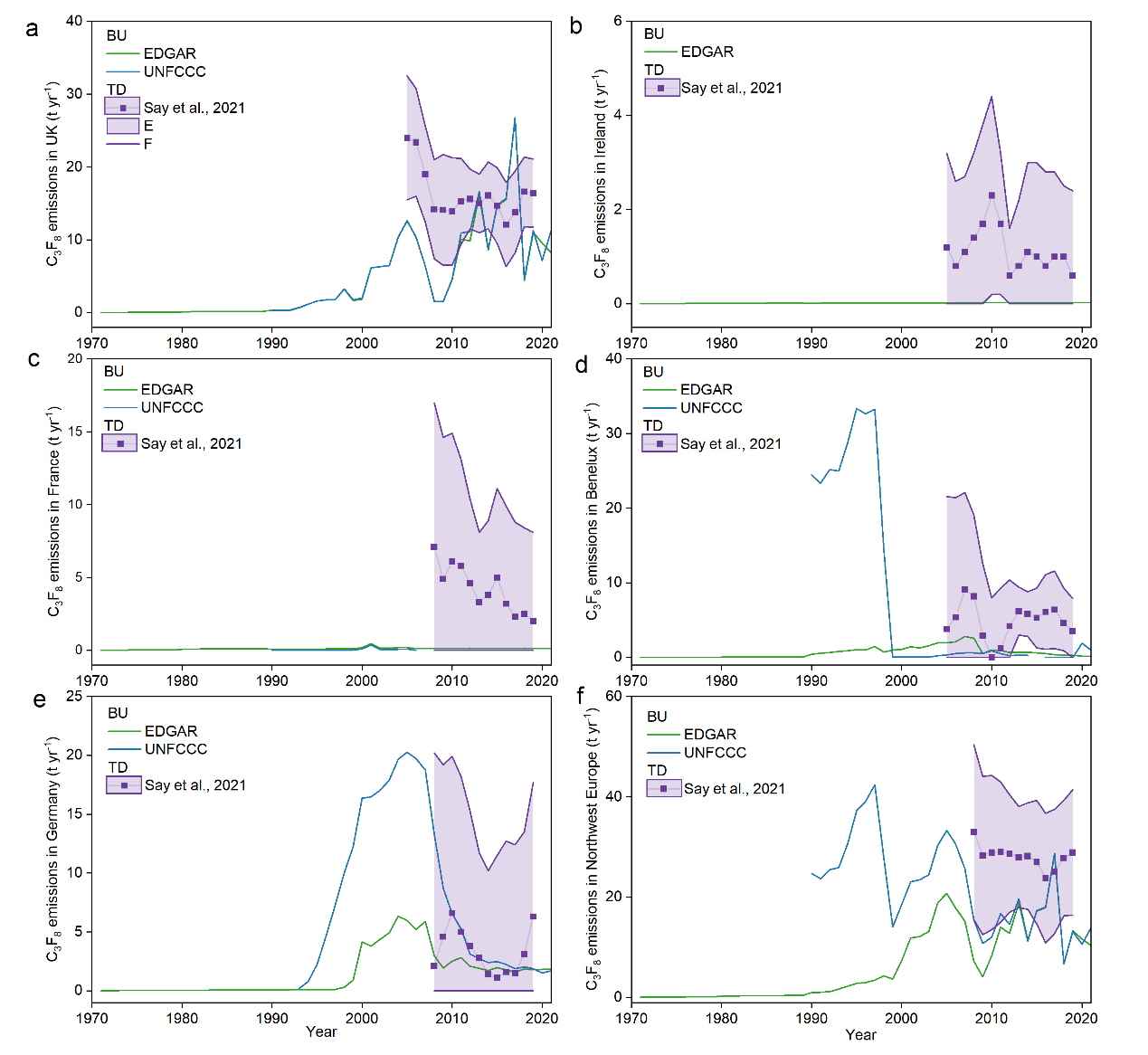


**Supplementary Figure 7**. Summary of TD and BU C_3_F_8_ emissions in Northwest Europe from previous studies for (**a**) UK, (**b**) Ireland, (**c**) France, (**d**) Benelux, (**e**) Germany, and (**f**) total Northwest Europe. The detailed sources can be found in Supplementary Table 3.


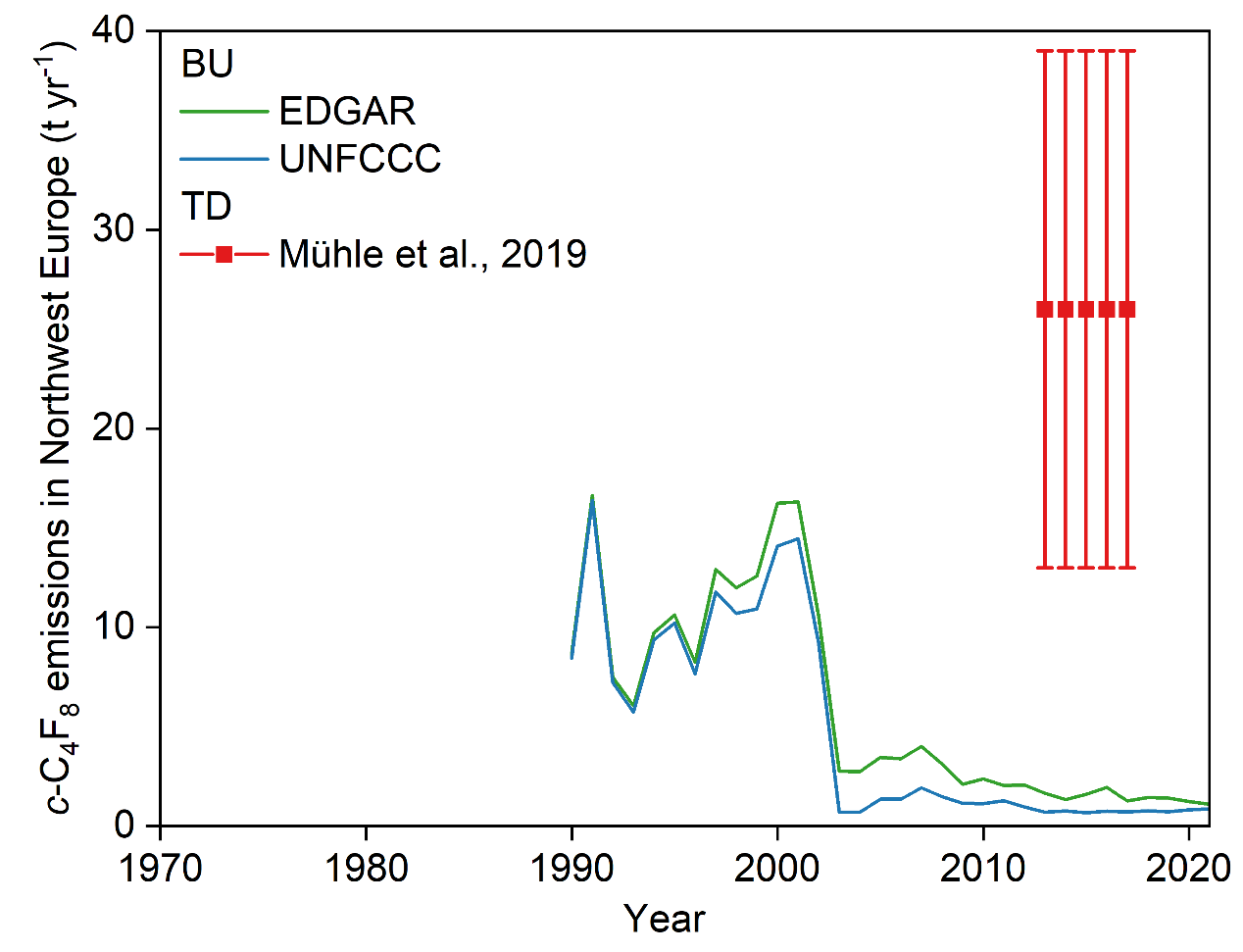


**Supplementary Figure 8**. Summary of TD and BU *c*-C_4_F_8_ emissions in Northwest Europe from previous studies. The detailed sources can be found in Supplementary Table 3.


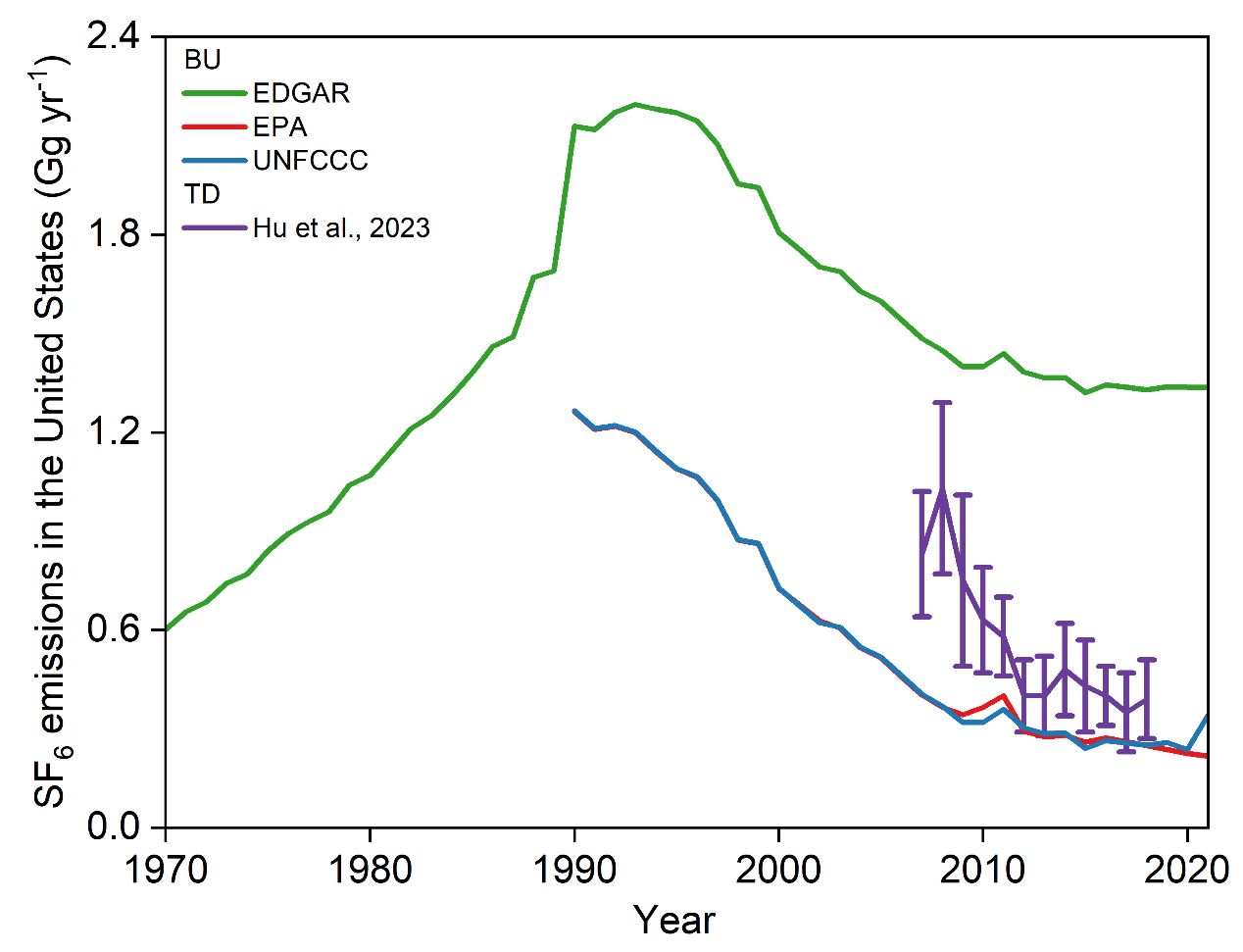


**Supplementary Figure 9**. Summary of TD and BU SF_6_ emissions in the United States from previous studies. The detailed sources can be found in Supplementary Table 3.


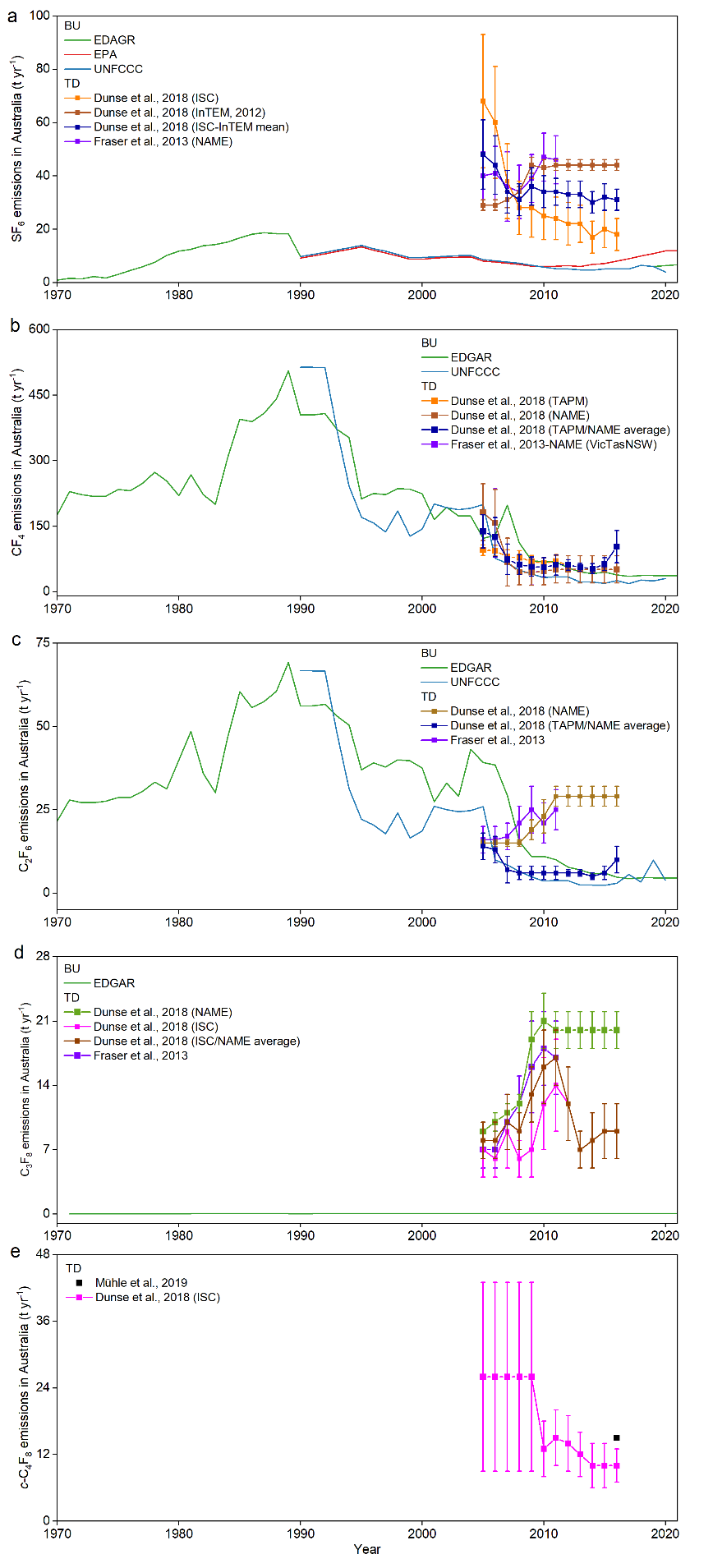


**Supplementary Figure 10**. Summary of TD and BU FFGHG emissions in Australia from previous studies for (**a**) SF_6_, (**b**) CF_4_, (**c**) C_2_F_6_, (**d**) C_3_F_8_, and (**e**) *c*-C_4_F_8_. The detailed sources can be found in Supplementary Table 3.


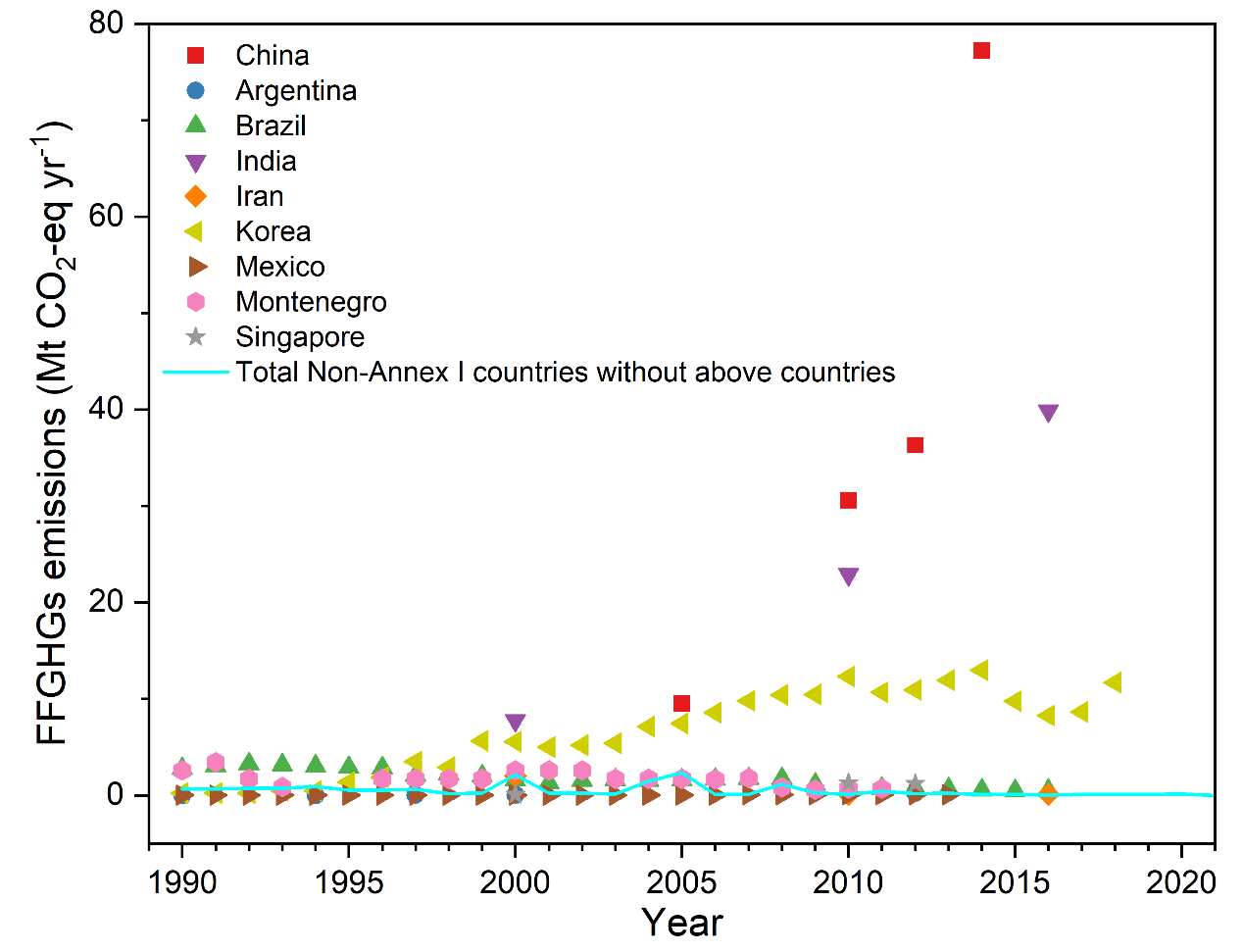


**Supplementary Figure 11**. FFGHG emissions for China, Argentina, Brazil, India, Iran, Korea, Mexico, Montenegro, Singapore, and the rest non-Annex I countries. Emissions are obtained from UNFCCC (<https://di.unfccc.int/flex_non_annex1>).

**Supplementary Tables**

**Supplementary Table 1. The global warming potentials over a 100-year horizon (GWP_100_) and lifetimes of fully fluorinated greenhouse gases in this work**

| Chemical  Name | Chemical Formula | IPCC AR4 (2007) | |
| --- | --- | --- | --- |
|  |  | GWP_100_ | Lifetime (yr) |
| Sulfur Hexafluoride | SF_6_ | 22800 | 3200 |
| Nitrogen Trifluoride | NF_3_ | 17200 | 740 |
| Perfluoromethane (PFC-14) | CF_4_ | 7390 | 50000 |
| Perfluoroethane (PFC-116) | C_2_F_6_ | 12200 | 10000 |
| Perfluoropropane (PFC-218) | C_3_F_8_ | 8830 | 2600 |
| Perfluorocyclobutane (PFC-318) | *c*-C_4_F_8_ | 10300 | 3200 |

Note: AR4 means Fourth Assessment Report^1^

**Supplementary Table 2. Summary of previous studies reporting TD global emissions for the single FFGHG**

| Studies | Regions | FFGHGs | Periods |
| --- | --- | --- | --- |
| WMO, 2022^2^ | Global | SF_6_&NF_3_&CF_4_&C_2_F_6_&C_3_F_8_&*c*-C_4_F_8_ | 1980–2021 |
| Simmonds et al., 2020^3^ | Global | SF_6_ | 1978–2018 |
| Levin et al., 2010^4^ | Global | SF_6_ | 1978–2008 |
| Rigby et al., 2010^5^  Arnold et al., 2013^6^ | Global  Global | SF_6_  NF_3_ | 1970–2008  1980–2011 |
| Say et al., 2021^7^ | Global | CF_4_&C_2_F_6_&C_3_F_8_ | 2005–2019 |
| Trudinger et al., 2016 | Global | CF_4_&C_2_F_6_&C_3_F_8_ | 1900–2014 |
| Mühle et al., 2010^8^ | Global | CF_4_&C_2_F_6_&C_3_F_8_ | 1975–2008 |
| Worton et al., 2007^9^ | Global | CF_4_&C_2_F_6_ | 1975–1999 |
| Khalil et al. 2003^10^ | Global | CF_4_&C_2_F_6_ | 1975–1999 |
| Harnisch et al., 1999^11^ | Global | CF_4_ | 1978–1990 & 1992–1998 |
| Harnisch et al., 1996^12^ | Global | CF_4_&C_2_F_6_ | 1982–1996 |
| Harnisch et al., 1995^13^ | Global | CF_4_&C_2_F_6_ | 1978–1995 |
| Culbertson et al., 2004^14^ | Global | C_3_F_8_ | 1977–1997 |
| Khalil et al. 2003^10^ | Global | C_3_F_8_ | 1978–1986 & 1994–1997 |
| Mühle et al., 2022^15^ | Global | *c*-C_4_F_8_ | 1990.5–2020.5 |
| Droste et al., 2020^16^ | Global | *c*-C_4_F_8_ | 1978–2017 |
| Mühle et al., 2019^17^ | Global | *c*-C_4_F_8_ | 1900–2017 & 1973–2017 |
| Oram, et al., 2012^18^ | Global | *c*-C_4_F_8_ | 1986–2005 |

Note: the detailed global emission data for the single FFGHG are shown in Figure 1 in the main text.

**Supplementary Table 3. Summary of previous studies reporting TD regional emissions for the single FFGHG**

| Studies | Regions | Countries | FFGHGs | Periods |
| --- | --- | --- | --- | --- |
| Hu et al. 2023^19^ | North America | United States | SF_6_ | 2007–2018 |
| Simmonds et al., 2020^3^ | West Europe | United Kingdom, Ireland, Benelux (Belgium, the Netherlands, and Luxembourg), Germany, France, Denmark, Switzerland, Austria, Spain, Italy, and Portugal | SF_6_ | 2007–2018 |
| Say et al. 2021^7^ | Northwest Europe | United Kingdom; Ireland; France; Belgium, the Netherlands, and Luxembourg (collectively termed Benelux); and Germany | CF_4_&C_2_F_6_&C_3_F_8_ | 2005–2019 |
| Mühle et al., 2019^17^ | Northwestern Europe | Ireland, United Kingdom, France, Germany, Belgium, the Netherlands, Luxemburg, and Denmark | *c*-C_4_F_8_ | 2013–2017 |
| Simmonds et al., 2020^3^ | Eastern Asia | China, South Korea | SF_6_ | 2007–2018 |
| Fang et al., 2014^20^ | Eastern Asia | Mongolia, China, North Korea, South Korea, Japan | SF_6_ | 2006–2012 |
| Vollmer et al., 2009^21^ | Eastern Asia | China | SF_6_ | 2006.10–2008.03 |
| Kim et al., 2021^22^  Mühle et al., 2019^17^ | Eastern Asia  Eastern Asia | China, Japan, Korea  Eastern China, Western Japan, South Korea, North Korea | CF_4_&C_2_F_6_  *c*-C_4_F_8_ | 2008–2019  2010–2017 |
| Arnold et al., 2018^23^ | Eastern Asia | China, South Korea, North Korea, Japan | CF_4_&NF_3_ | 2008–2015(CF4) & 2014–2015(NF3) |
| Yao et al., 2012^24^ | Eastern Asia | China | CF_4_&C_2_F_6_&C_3_F_8_ | 2020.5–2011.5 |
| Kim et al. 2011^25^ | Eastern Asia | China, Japan, Korea | SF_6_&CF_4_&C_2_F_6_&C_3_F_8_ | 2007.11–2008.12 |
| Satio et al., 2010^26^ | Eastern Asia | China, South Korea, North Korea, Japan | C_2_F_6_&C_3_F_8_&*c*-C_4_F_8_ | 2007.11–2009.09 |
| Kim et al., 2010^27^ | Eastern Asia | China | SF_6_&CF_4_&C_2_F_6_&C_3_F_8_ | 2008 |
| Yokouchi et al., 2005^28^ | Eastern Asia | Japan | SF_6_&C_2_F_6_&C_3_F_8_&*c*-C_4_F_8_ | 2003 |
| Mühle et al., 2019^17^ | Oceania | Southeastern Australia | *c*-C_4_F_8_ | 2016 |
| Dunse et al., 2018^29^ | Oceania | Australia | SF_6_&CF_4_&C_2_F_6_&C_3_F_8_&*c*-C_4_F_8_ | 2005–2016 |
| Fraser et al., 2013^30^ | Oceania | Australia | SF_6_&CF_4_&C_2_F_6_&C_3_F_8_ | 2005–2011 |
| Mühle et al., 2019^17^ | Asia | Northern and central India (NCI) | *c*-C_4_F_8_ | 2016.06–2016.07 |
| Mühle et al., 2019^17^ |  | Russia | *c*-C_4_F_8_ | 2016 |

**Supplementary References**

1. Forster P*, et al.* Changes in Atmospheric Constituents and in Radiative Forcing. In: Climate Change 2007: The Physical Science Basis. Contribution of Working Group I to the Fourth Assessment Report of the Intergovernmental Panel on Climate Change. [Solomon, S., D. Qin, M. Manning, Z. Chen, M. Marquis, K.B. Averyt, M.Tignor and H.L. Miller] edn. Cambridge University Press, Cambridge, United Kingdom and New York, NY, USA. (2007).

2. Johannes Laube and Susann Tegtmeier (Leaing Authors)*, et al.* Update on Ozone-Depleting Substances (ODSs) and Other Gases of Interest to the Montreal Protocol, Chapter 1 in Scientific Assessment of Ozone Depletion: 2022. GAW Report No. 278, 509 pp., WMO, Geneva, 2022.

3. Simmonds PG*, et al.* The increasing atmospheric burden of the greenhouse gas sulfur hexafluoride (SF_6_). *Atmos. Chem. Phys.* **20**, 7271-7290 (2020).

4. Levin I*, et al.* The global SF_6_ source inferred from long-term high precision atmospheric measurements and its comparison with emission inventories. *Atmospheric Chemistry and Physics* **10**, 2655-2662 (2010).

5. Rigby M*, et al.* History of atmospheric SF_6_ from 1973 to 2008. *Atmos. Chem. Phys.* **10**, 10305-10320 (2010).

6. Arnold T*, et al.* Nitrogen trifluoride global emissions estimated from updated atmospheric measurements. *Proceedings of the National Academy of Sciences* **110**, 2029 (2013).

7. Say D*, et al.* Global trends and European emissions of tetrafluoromethane (CF_4_), hexafluoroethane (C_2_F_6_) and octafluoropropane (C_3_F_8_). *Atmos. Chem. Phys.* **21**, 2149-2164 (2021).

8. Mühle J*, et al.* Perfluorocarbons in the global atmosphere: tetrafluoromethane, hexafluoroethane, and octafluoropropane. *Atmospheric Chemistry and Physics* **10**, 5145-5164 (2010).

9. Worton DR*, et al.* Atmospheric trends and radiative forcings of CF4 and C2F6 inferred from firn air. *Environ. Sci. Technol.* **41**, 2184-2189 (2007).

10. Aslam MK, K., Rasmussen RA, Culbertson JA, Prins JM, Grimsrud EP, Shearer MJ. Atmospheric perfluorocarbons. *Environ. Sci. Technol.* **37**, 4358-4561 (2003).

11. Harnisch J, Borchers R, Fabian P, Maiss M. CF4 and the Age of Mesospheric and Polar Vortex Air. *Geophys. Res. Lett.* **26**, 295-298 (1999).

12. Harnisch J, Borchers R, Fabian P, Maiss M. Tropospheric trends for CF4 and C2F6 since 1982 derived from SF6 dated stratospheric air. *Geophys. Res. Lett.* **23**, 1099-1102 (1996).

13. Harnisch J, Borchers R, Fabian P. Estimation of tropospheric trends (1980 - 1995) for CF4 and C2F6 from stratospheric data. (1995).

14. Culbertson JA, Prins JM, Grimsrud EP, Rasmussen RA, Khalil MA, Shearer MJ. Observed trends for CF3-containing compounds in background air at Cape Meares, Oregon, Point Barrow, Alaska, and Palmer Station, Antarctica. *Chemosphere* **55**, 1109-1119 (2004).

15. Mühle J*, et al.* Global Emissions of Perfluorocyclobutane (PFC-318, *c*-C_4_F_8_) Resulting from the Use of Hydrochlorofluorocarbon-22 (HCFC-22) Feedstock to Produce Polytetrafluoroethylene (PTFE) and related Fluorochemicals. *Atmospheric Chemistry and Physics*, (2022).

16. Droste ES*, et al.* Trends and emissions of six perfluorocarbons in the Northern Hemisphere and Southern Hemisphere. *Atmos. Chem. Phys.* **20**, 4787-4807 (2020).

17. Mühle J*, et al.* Perfluorocyclobutane (PFC-318, *c*-C_4_F_8_) in the global atmosphere. *Atmos. Chem. Phys.* **19**, 10335-10359 (2019).

18. Oram DE*, et al.* Long-term tropospheric trend of octafluorocyclobutane (*c*-C_4_F_8_ or PFC-318). *Atmos. Chem. Phys.* **12**, 261-269 (2012).

19. Hu L*, et al.* Declining, seasonal-varying emissions of sulfur hexafluoride from the United States. *Atmospheric Chemistry and Physics* **23**, 1437-1448 (2023).

20. Fang X*, et al.* Sulfur hexafluoride (SF_6_) emissions in East Asia determined by inverse modeling. *Atmos. Chem. Phys.* **14**, 4779-4791 (2014).

21. Vollmer MK*, et al.* Emissions of ozone-depleting halocarbons from China. *Geophys. Res. Lett.* **36**, L15823 (2009).

22. Kim J*, et al.* Emissions of tetrafluoromethane (CF_4_) and hexafluoroethane (C_2_F_6_) from East Asia: 2008 to 2019. *Journal of Geophysical Research: Atmospheres* **126**, e2021JD034888 (2021).

23. Arnold T*, et al.* Inverse modelling of CF_4_ and NF_3_ emissions in East Asia. *Atmos. Chem. Phys.* **18**, 13305-13320 (2018).

24. Yao B*, et al.* In-situ measurements of atmospheric hydrofluorocarbons (HFCs) and perfluorocarbons (PFCs) at the Shangdianzi regional background station, China. *Atmos. Chem. Phys.* **12**, 10181-10193 (2012).

25. Li S*, et al.* Emissions of halogenated compounds in East Asia determined from measurements at Jeju Island, Korea. *Environ. Sci. Technol.* **45**, 5668-5675 (2011).

26. Saito T, Yokouchi Y, Stohl A, Taguchi S, Mukai H. Large emissions of perfluorocarbons in East Asia deduced from continuous atmospheric measurements. *Environ. Sci. Technol.* **44**, 4089-4095 (2010).

27. Kim J*, et al.* Regional atmospheric emissions determined from measurements at Jeju Island, Korea: Halogenated compounds from China. *Geophys. Res. Lett.* **37**, L12801 (2010).

28. Yokouchi Y, Inagaki T, Yazawa K, Tamaru T, Enomoto T, Izumi K. Estimates of ratios of anthropogenic halocarbon emissions from Japan based on aircraft monitoring over Sagami Bay, Japan. *Journal of Geophysical Research: Atmospheres* **110**, (2005).

29. Dunse BL, Derek N, Fraser PJ, Krummel PB, Steele LP. Australian and global HFC, PFC, Sulfur Hexafluoride, Nitrogen Trifluoride and Sulfuryl Fluoride Emissions. Australian Government Department of the Environment and Energy (2018).

30. Fraser P, Dunse B, Krummel P, Steele P, Derek N. Australian Atmospheric Measurements & Emissions Estimates of Ozone Depleting Substances and Synthetic Greenhouse Gases Report prepared for Department of the Environment. CSIRO Marine and Atmospheric Research, Centre for Australian Weather and Climate Research, Aspendale, Australia (2013).
